# Essential Role for miR-196a in Brown Adipogenesis of White Fat Progenitor Cells

**DOI:** 10.1371/journal.pbio.1001314

**Published:** 2012-04-24

**Authors:** Masaki Mori, Hironori Nakagami, Gerardo Rodriguez-Araujo, Keisuke Nimura, Yasufumi Kaneda

**Affiliations:** 1 Division of Gene Therapy Science, Graduate School of Medicine, Osaka University, Osaka, Japan; 2 Division of Vascular Medicine and Epigenetics, United Graduate School of Child Development, Osaka University, Osaka, Japan; University of Cambridge, United Kingdom

## Abstract

The recent discovery of functional brown adipocytes in adult humans illuminates the potential of these cells in the treatment of obesity and its associated diseases. In rodents, brown adipocyte-like cells are known to be recruited in white adipose tissue (WAT) by cold exposure or β-adrenergic stimulation, but the molecular machinery underlying this phenomenon is not fully understood. Here, we show that inducible brown adipogenesis is mediated by the microRNA miR-196a. We found that miR-196a suppresses the expression of the white-fat gene *Hoxc8* post-transcriptionally during the brown adipogenesis of white fat progenitor cells. In mice, miR-196a is induced in the WAT-progenitor cells after cold exposure or β-adrenergic stimulation. The fat-specific forced expression of miR-196a in mice induces the recruitment of brown adipocyte-like cells in WAT. The miR-196a transgenic mice exhibit enhanced energy expenditure and resistance to obesity, indicating the induced brown adipocyte-like cells are metabolically functional. Mechanistically, Hoxc8 targets and represses *C/EBPβ*, a master switch of brown-fat gene program, in cooperation with histone deacetylase 3 (HDAC3) through the *C/EBPβ* 3′ regulatory sequence. Thus, miR-196a induces functional brown adipocytes in WAT through the suppression of *Hoxc8*, which functions as a gatekeeper of the inducible brown adipogenesis. The miR-196a-*Hoxc8*-*C/EBPβ* signaling pathway may be a therapeutic target for inducing brown adipogenesis to combat obesity and type 2 diabetes.

## Abstract 2

Brown adipocytes can differentiate from white fat progenitor cells in mice exposed to cold or β3-adrenergic stimulation, and this process is regulated by a microRNA that regulates the expression of Hoxc8, a master regulator of brown adipogenesis.

## Author Summary

Obesity is caused by the accumulation of surplus energy in a fatty tissue called white adipose tissue (WAT) and can lead to important health problems such as diabetes. Mammals additionally possess brown adipose tissue (BAT), which serves to generate body heat to stabilize body temperature under exposure to cold, and is abundant in hibernating animals and human neonates. In performing its function BAT consumes energy, thereby reducing WAT fat accumulation. Recent studies have shown that exposure to a cold environment stimulates the partial conversion of WAT to BAT in mice, and given that human adults have a limited amount of BAT, such a conversion has the potential to afford a novel method of obesity control. Here, we analyze the molecular mechanism of this conversion using genetically manipulated mice and cells isolated from human adipose tissue. We find that the expression levels of a microRNA, miR-196a, positively correlate with the conversion of WAT to BAT under cold exposure conditions. We show that forced expression of miR-196a in mouse adipose tissue increases BAT content and energy expenditure, thereby rendering the animals resistant to obesity and diabetes. Mechanistically, we observe that miR-196a acts by inhibiting the expression of the homeotic gene Hoxc8, a repressor of brown adipogenesis. These findings introduce the therapeutic possibility of using microRNAs to control obesity and its associated diseases in humans.

## Introduction

Brown adipose tissue (BAT) combusts excess energy through mitochondrial energy uncoupling mediated by Uncoupling protein-1 (Ucp1, also known as thermogenin) in nonshivering thermogenesis [1]. Recent discoveries of metabolically active BAT in adult humans [2]–[6] have highlighted BAT as a new therapeutic target for treating obesity and its associated diseases, such as type 2 diabetes mellitus [7]. The activity of BAT is inversely correlated with body mass index in humans [3]–[4], implying a significant role for BAT in the development of obesity. Importantly, the brown adipocyte-like cells in white adipose tissue (WAT) can be generated by cold exposure or β3-adrenergic stimulation in rodents [8]–[9], and the activity of BAT can be increased by cold exposure or β3-adrenergic stimulation in humans [2]. The molecular mechanisms underlying this inducible brown adipogenesis have not been fully elucidated.

The expression patterns of the *Hox* family of homeobox genes (Hox genes) are characteristically distinct between BAT and WAT [10]–[12], which implies a significant role of Hox genes in the determination of two fat types. But its significance has not been fully understood. Hox genes are representative of developmental genes and confer an anteroposterior positional identity during embryogenesis. Several Hox genes have roles in differentiation systems, such as hematopoiesis [13], myogenesis [14], and cardiogenesis [15], but relatively less is known about their roles in adipogenesis. Among the differentially expressed Hox genes, *Hoxc8* is more highly expressed in WAT than in BAT and is categorized as a white-fat gene [11],[16]. These observations imply that *Hoxc8* may have an unknown role in the determination of the two fat types.

microRNAs (miRNAs) are important regulators of the gene networks underlying diverse biological phenomena [17]. miRNAs are small, non-coding RNAs that base pair with specific mRNAs and suppress gene expression post-transcriptionally [18]. miRNAs constitute an essential regulatory layer at the level of the transcriptional network [19]. Through their regulatory capacity, miRNAs affect the output of signaling networks by fine-tuning or switching output levels [19] and promote or redirect dynamic flow in genetic circuits and affect differentiation [20]. The roles of miRNAs in the inducible brown adipogenesis in WAT are not well understood.

We here show that single miRNA miR-196a is capable of recruiting the metabolically functional brown adipocytes in WAT in mice. The miR-196a expression is induced in the WAT-progenitor cells in mice exposed to cold or β3-adrenergic stimulation. The induction of miR-196a is required for the brown fat gene expression and is sufficient to generate the metabolically functional brown adipocyte-like cells in WAT in mice. The target gene of miR-196a is white-fat gene *Hoxc8*, which directly represses the expression of *C/EBPβ*, a master regulator of brown adipogenesis.

## Results

### *HOXC8* Represses Brown-Fat Genes and Is Down-Regulated During Brown Adipogenesis of Human WAT-Progenitor Cells

**Figure 1:**
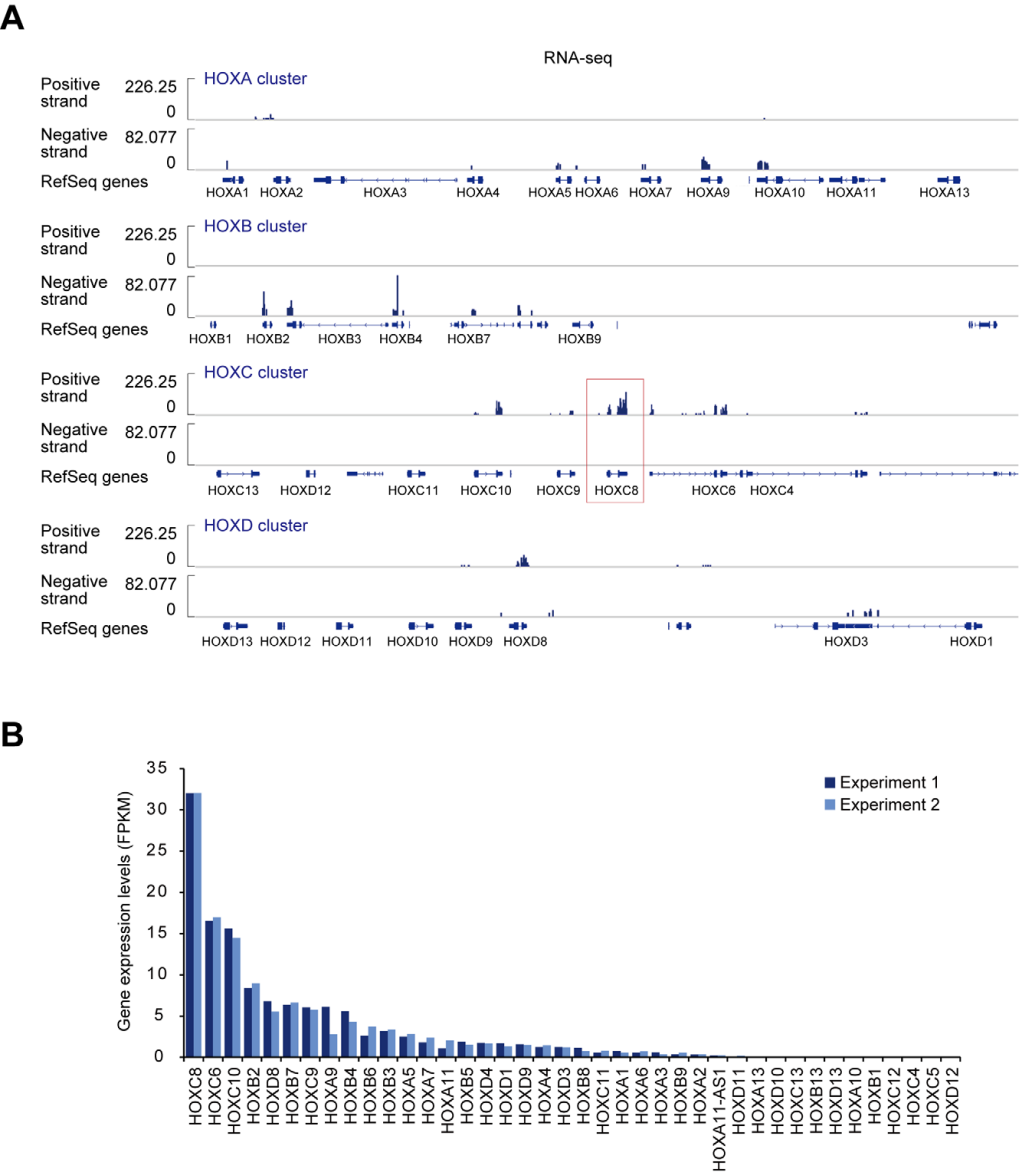
*HOXC8* is most highly expressed among clustered HOX genes in human WAT-progenitor cells. (A) Strand-specific RNA-seq results showing the expression levels of clustered HOX genes in undifferentiated human white fat (WAT) progenitor cells. The results with the clusters of HOXA, HOXB, HOXC, and HOXD are shown. The position of RefSeq genes are shown below. (B) The expression levels of clustered Hox genes from two biological replicates. FPKM, fragments per kilobase of exon per million mapped fragments.

**Figure 2:**
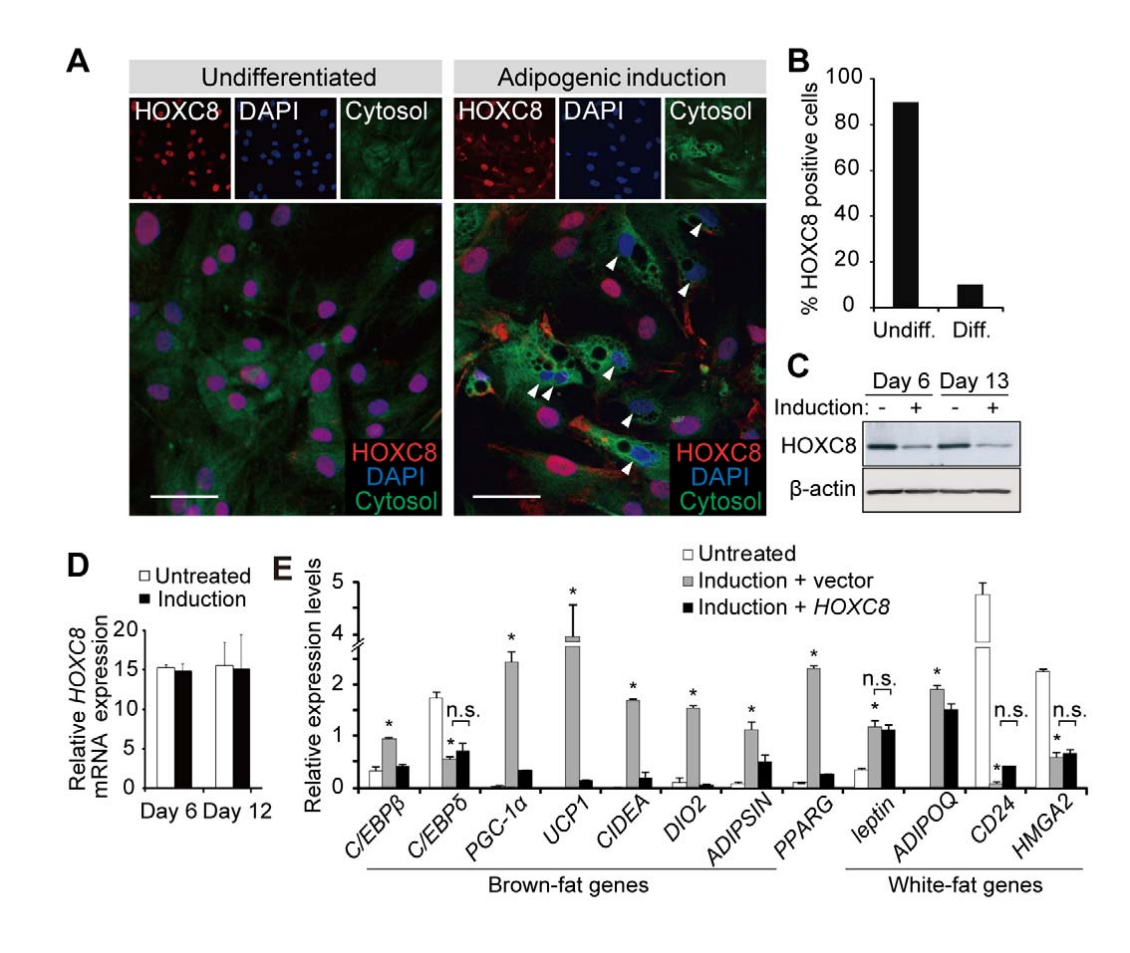
HOXC8 represses brown-fat genes and is down-regulated during brown adipogenesis of human WAT-progenitor cells. (A) The immunofluoresence analysis of HOXC8 in human WAT-progenitor cells. Arrowheads indicate differentiated adipocytes that exhibit multiple vesicles. The cells were counterstained with CellTracker and DAPI. The scale bars indicate 30 µm. (B) The percentage of HOXC8-expressing cells among undifferentiated cells (Undiff) and differentiated cells (Diff). (C) The immunoblots for HOXC8 in human WAT-progenitor cells treated with an adipogenic induction medium (Induction) or left untreated. β-actin was used as a loading control. (D) The qRT-PCR of *HOXC8* mRNA expression levels in human WAT-progenitor cells left untreated or induced to undergo differentiation. The data were normalized to *18S*. (E) The qRT-PCR analysis of genes in human WAT-progenitor cells transduced with *Hoxc8* or control vector and induced to undergo differentiation. The results are normalized to *18S*. All data are presented as means ± SEM; * *p*<0.05 versus untreated. n.s., not significant.

Recent reports have shown that the WAT-derived progenitor cells undergo brown adipogenesis in vitro in both mice [16],[21] and humans [16],[22]. Consistently, the human fat progenitor cells derived from flank subcutaneous WAT (hereafter, WAT-progenitor cells) exhibited increased brown-fat gene expression after differentiation (Figure S1A and S1B). *HOXC8* is categorized as a white-fat gene [16] and RNA-seq analysis revealed that *HOXC8* was most highly expressed among the clustered HOX genes in the human WAT-progenitor cells (Figure 1A and 1B). We noticed that HOXC8 was down-regulated in the differentiated adipocytes (Figure 2A and 2B). Contrarily, the expression of HOXC6 did not change after differentiation (Figure S1C) and was not particularly high in WAT (Figure S1D), though *HOXC6* is located adjacent to *HOXC8* in HOXC cluster and was the second most highly expressed gene (Figure 1A and 1B). These results implied the existence of specific regulatory machinery for HOXC8 expression. Down-regulation of HOXC8 was observed at the protein level (Figure 2C) but not at the mRNA level (Figure 2D). These results implied that HOXC8 might be regulated post-transcriptionally. Transduction of *HOXC8* in the human WAT-progenitor cells suppressed the brown-fat genes including *C/EBPβ* [23], *UCP1* [24], and *ADIPSIN* (also known as *CFD*) (Figure 2E) [23]. In contrast, *HOXC8* did not suppress the white-fat genes including *leptin* [11], *CD24* [25], *HMGA2* [26], and *ADIPOQ* (also called *adiponectin*) (Figure 2E). These results suggested that *HOXC8* might regulate the brown-fat genes and that HOXC8 might be an important regulator for brown adipogenesis of the WAT-progenitor cells.

### *Hoxc8* Is Down-Regulated During Brown Adipogenesis In Vivo

**Figure 3:**
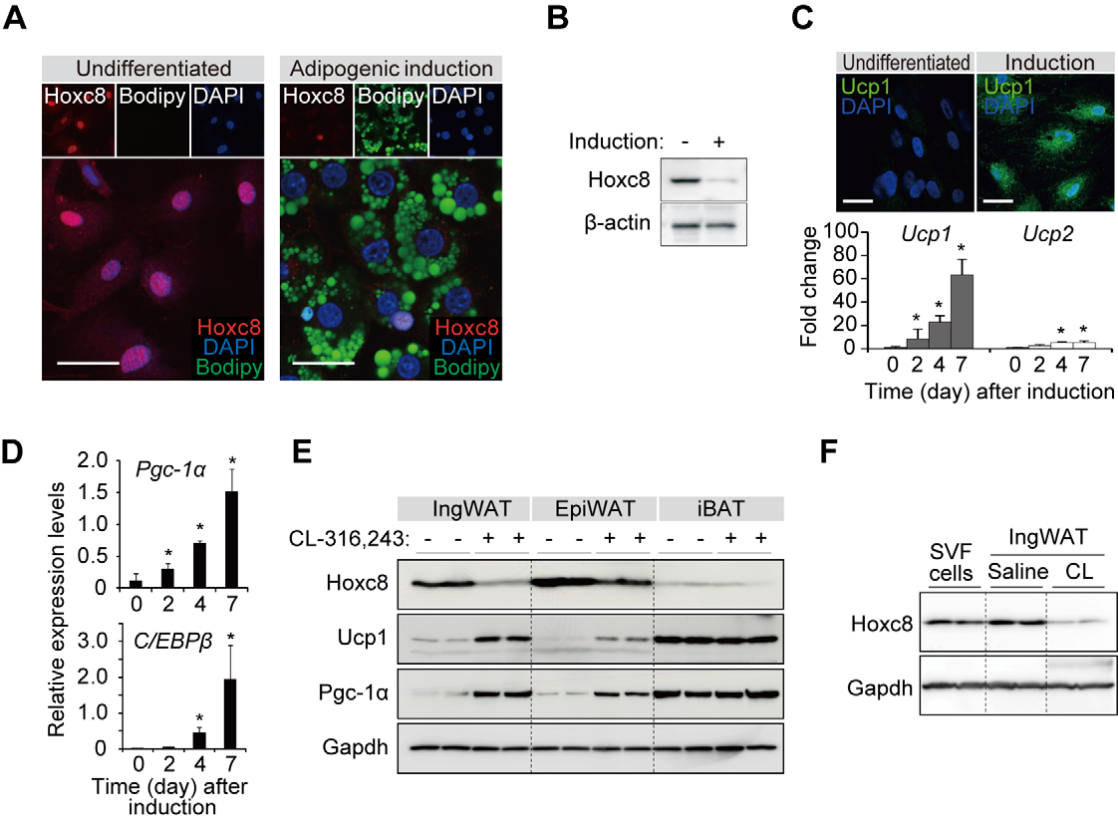
Hoxc8 is down-regulated during brown adipogenesis in vivo. (A) Immunofluorescence analysis of Hoxc8 in the mouse SVF cells derived from inguinal WAT. The cells were untreated (Undifferentiated) or induced to undergo differentiation (Adipogenic induction). The lipid droplets and nuclei were counterstained with Bodipy and DAPI, respectively. Scale bars indicate 30 µm. (B) Immunoblots of Hoxc8 in the mouse SVF cells left untreated or induced to undergo differentiation. β-actin served as a loading control. (C) Upper, the UCP1 expression in the differentiated mouse SVF cells. Scale bars indicate 30 µm. Lower, the fold increase of mRNA expression levels of *Ucp1* and *Ucp2* in the mouse WAT-SVF cells induced to undergo differentiation. The results were normalized to *β-actin*. (D) The expression of *Pgc-1α* and *C/EBPβ* induced during the differentiation of mouse SVF cells. The results were normalized to *β-actin*. The data are presented as means ± SEM; * *p*<0.05. (E) Western blot analysis in different fat depots from mice treated with or without CL-316,243, a β3-adrenergic receptor agonist. ingWAT, epiWAT, and iBAT denote inguinal WAT, epididymal WAT, and interscapular BAT, respectively. (F) Western blot analysis of Hoxc8 in SVF cells and ingWAT of mice treated with CL-316,243 (CL) or saline.

To extend our findings in vitro to in vivo, we proceeded to a mouse model of brown adipogenesis. In mice, the *Hoxc8* expression was higher in WAT than BAT and other tissues (Figure S2). Stromal vascular fraction (SVF) of fat depots contains fat progenitor cells (hereafter, SVF cells). The Hoxc8 expression was suppressed after the SVF cells were induced to undergo brown adipogenesis (Figure 3A and 3B) and expressed Ucp1 (Figure 3C), *Pgc-1α*, and *C/EBPβ* (Figure 3D). In mice, brown adipogenesis can be induced in WAT by administering a β3-adrenergic agonist, CL-316,243, or by exposing mice to cold environment. After administration of CL-316,243, the expression of Hoxc8 was down-regulated prominently in inguinal WAT (ingWAT) (Figure 3E). The down-regulation of Hoxc8 was relatively modest in epididymal WAT (epiWAT) and interscapular BAT (iBAT) than in ingWAT (Figure 3E). To delineate the Hoxc8 expression changes during white and brown adipogenesis, the Hoxc8 expression levels were compared between the progenitor cell fraction (SVF) and tissue fraction mainly composed of mature adipocytes. As a result, the Hoxc8 expression is slightly increased in saline-treated WAT than in SVF and is down-regulated in CL-316,243-treated fat that underwent brown adipogenesis, indicating that Hoxc8 is down-regulated specifically during brown adipogenesis, but not during white adipogenesis (Figure 3F). Thus, the down-regulation of Hoxc8 is observed during brown adipogenesis both in vitro and in vivo.

### *miR-196a* Regulates *Hoxc8* Expression in Brown Adipogenesis of WAT-Progenitor Cells

**Figure 4:**
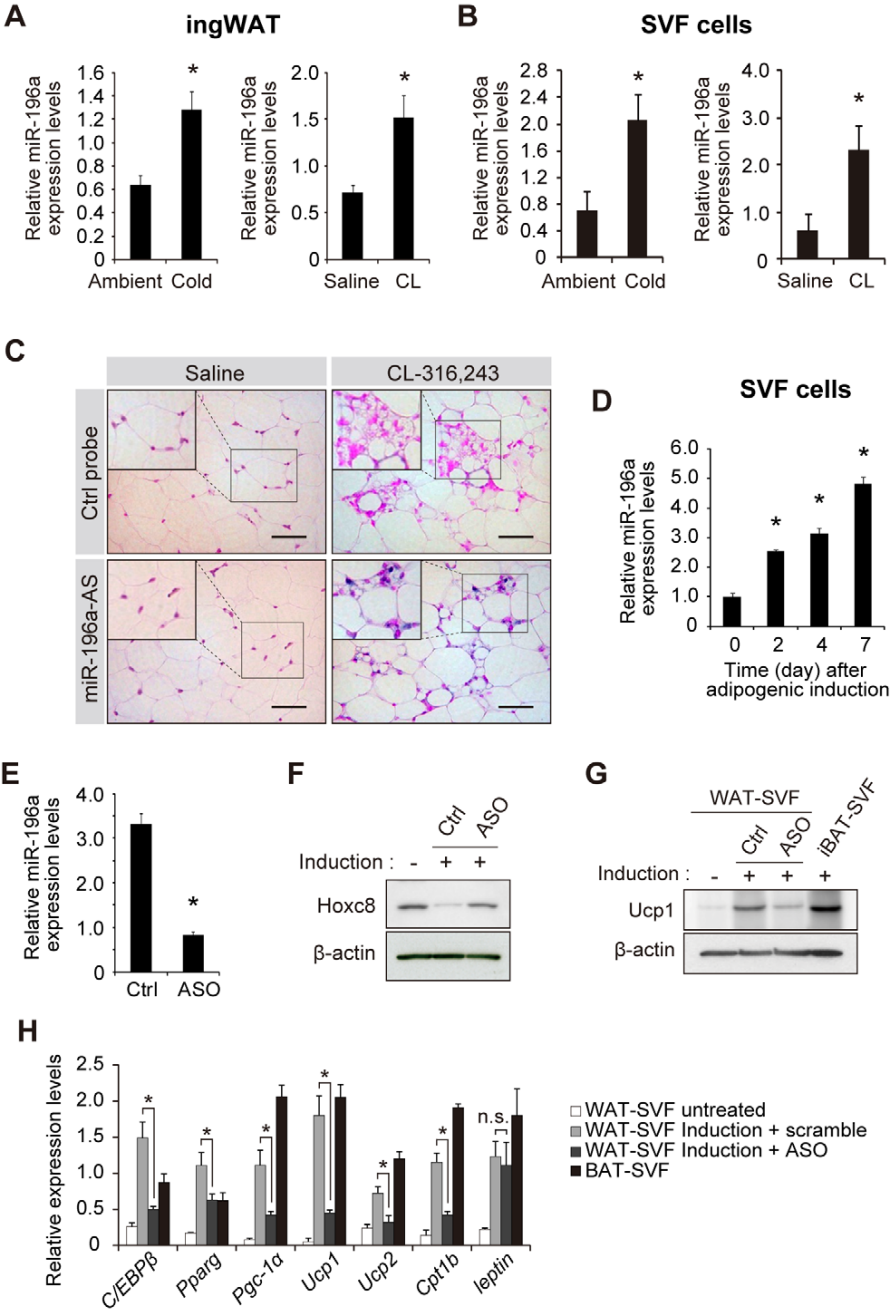
miR-196a is induced in SVF cells during brown adipogenesis and is required for *UCP1* expression. (A) The expression of miR-196a in the ingWAT depots of mice housed at 4°C (Cold) or at ambient temperature for 5 h (*n* = 6), and of mice treated with CL-316,243 (CL) or saline for 7 consecutive days (*n* = 6). The results are normalized to *U6*. (B) The expression of miR-196a in WAT-SVF cells of the mice exposed to cold environment or CL-316,243 (CL, *n* = 6). The data are normalized to *U6*. (C) The in situ hybridization of miR-196a in the ingWAT depots of mice treated with CL-316,243 or saline. The sections were probed with a miR-196a-antisense (AS) probe or control (Ctrl) probe. Size bars indicate 50 µm. All data are presented by means ± SEM; * *p*<0.05. (D) The miR-196a expression levels in mouse SVF cells during differentiation in vitro. (E) The miR-196a expression level in the mouse SVF cells transfected with antisense oligonucleotides (ASO) against miR-196a. (F) The immunoblots for Hoxc8 in mouse SVF cells transfected with ASO against miR-196a or with control (Ctrl) oligonucleotides after differentiation. β-actin served as a loading control. (G) The immunoblots for Ucp1 in mouse SVF cells transfected with ASO against miR-196a or with Ctrl oligo followed by adipogenic induction. β-actin served as a loading control. (H) The mRNA expression levels in the mouse SVF cells transfected with ASO against miR-196a or control (Ctrl) oligonucleotides followed by differentiation induction. The results were normalized to *β-actin*. All data are presented by means ± SEM; * *p*<0.05.

We next sought to identify the mechanism underlying the down-regulation of Hoxc8 during brown adipogenesis. Post-transcriptional regulation of Hoxc8 was suggested by the in vitro experiments. Characteristically, a number of Hox genes are regulated by miRNAs [14],[27]–[29] and the Hoxc8 expression can be down-regulated by evolutionally conserved miR-196a via translational inhibition during vertebrate development [28]. There are two genes encoding miR-196a (miR-196a-1 and miR-196a-2) located within the Hox gene clusters [28]. Based on the hypothesis that Hoxc8 might be regulated by miR-196a, we investigated the miR-196a expression during the brown adipogenesis in mice. We found that the miR-196a expression was induced in WAT depots of mice exposed to cold environment or β3-adrenergic stimulations (Figure 4A). More specifically, miR-196a was more highly induced in the SVF cells (Figure 4B) than in mature adipocytes (Figure S3). Thus, miR-196a expression is induced in the SVF cells in mice exposed to β3-adrenergic stimulation or cold exposure. The in situ hybridization analysis of miR-196a showed the induction of miR-196a in WAT after CL-316,243 administration (Figure 4C). Based on the finding that the miR-196a expression is induced during the brown adipogenesis in WAT in mice, we next investigated whether the miR-196a induction is required for the induction of brown adipogenesis and Hoxc8 suppression. In vitro, the miR-196a expression is induced during the differentiation of WAT-progenitor cells derived from both mice (Figure 4D) and humans (Figures S4A). More detailed analyses showed that miR-196a was induced by forskolin, an adenylyl cyclase activator, implying the significant role of cyclic AMP pathway to regulate miR-196a expression (Figure S4B). To address the necessity of miR-196a in the brown adipogenesis, antisense oligonucleotide (ASO) against mR-196a was transfected to the mouse SVF cells. The miR-196a expression was suppressed in the transfected cells (Figure 4E) and the Hoxc8 expression was recovered in the transfected adipocytes (Figure 4F), indicating that Hoxc8 suppression was mediated by miR-196a. The ASO against miR-196a suppressed the expression of *Ucp1* (Figure 4G and 4H) and other brown-fat genes (Figure 4H), but not the *leptin* expression, indicating that miR-196a is necessary for the brown fat gene expression. Thus, the upregulation of miR-196a is required for the induction of brown fat gene expression during the differentiation of WAT-progenitor cells.

We next sought whether the findings above are possible to be generalized to the conventional brown adipogenesis, which occurs in the iBAT. The miR-196a expression level was significantly lower in iBAT than WAT (Figure S4C) and was not altered during the differentiation of the iBAT-SVF cells (Figure S4D), suggesting that miR-196a might not be involved in conventional brown adipogenesis in iBAT. Furthermore, endogenous expression of Hoxc8 was not detected in iBAT-SVF cells (Figure S5). Taken together, miR-196a is upregulated in the WAT-progenitor cells during the inducible brown adipogenesis in mice and is required for the induction of brown fat gene expression.

### *miR-196a* Induces Brown-Fat Genes Through *Hoxc8* Suppression

We next asked whether Hoxc8 was an essential target of miR-196a for the induction of brown-fat genes. We cloned the wild-type (Hoxc8-wt3′UTR) and miR-196a-binding site-deleted (Hoxc8-ΔmiR-196-BS) Hoxc8-3′UTR into a pCX4 retroviral vector and transduced these constructs into human WAT-progenitor cells (Figure S6A). The exogenous expression levels were comparable among the constructs (Figure S6A). After the adipogenic induction, the protein expression of Hoxc8 was suppressed in the Hoxc8-wt3′UTR-transduced cells than in Hoxc8-ΔmiR-196-BS- or Hoxc8-transduced cells (Figure S6B), suggesting that the suppression of Hoxc8 was dependent on the miR-196a-binding site in the Hoxc8 3′UTR. The brown fat gene expression was specifically high in the Hoxc8-wt3′UTR-tranduced cells (Figure S6C), indicating that the induction of brown-fat genes was regulated in a manner dependent on the miR-196a-binding site of Hoxc8 mRNA. These results suggest that miR-196a regulates brown-fat genes through suppression of Hoxc8. To further corroborate that Hoxc8 suppression is an important step, Hoxc8 was knocked down using Hoxc8 shRNA (Figure S7). As a result, the brown-fat genes including *C/EBPβ* and *Ucp1* were induced (Figure S7A and S7B), indicating that the suppression of Hoxc8 is a critical step for the induction of brown-fat genes.

### miR-196a Induces Brown Adipocyte-Like Cells in WAT

Based on the finding that miR-196a is required for the inducible brown adipogenesis, we next addressed whether miR-196a is capable of inducing brown adipogenesis in mice. We created transgenic mice in which miR-196a and EGFP were expressed under the control of the *aP2* promoter/enhancer, which is exclusively active in adipose tissues [30]. The transgenic mice (hereafter, the miR-196a mice) were born in a Mendelian ratio and were viable. The SVF cells isolated from the miR-196a mice were EGFP-negative immediately upon isolation, but they became EGFP-positive while they were kept in culture (Figure S8A) and expressed miR-196a (Figure S8B), resulting in Hoxc8 suppression (Figure S8C and S8D). After differentiation induction, the cells expressed more intense EGFP and underwent adipogenesis. The *aP2* promoter activity was observed in the fibroblast-like cells in ingWAT depots (Figure S8E), which might represent the fat progenitor cells undergoing adipogenesis. The SVF cells isolated from the miR-196a mice expressed brown-fat genes more highly than the cells from wild-type (WT) mice after differentiation in vitro (Figure S8F), indicating that miR-196a promotes brown adipocyte differentiation of the WAT-progenitor cells. To ask whether the miR-196a function is cell-autonomous, the human WAT-progenitor cells were transduced with lentivirus expressing miR-196a. As a result, miR-196a enhanced the brown fat gene expression during differentiation, indicating the cell-autonomous function of miR-196a (Figure S9).

**Figure 5:**
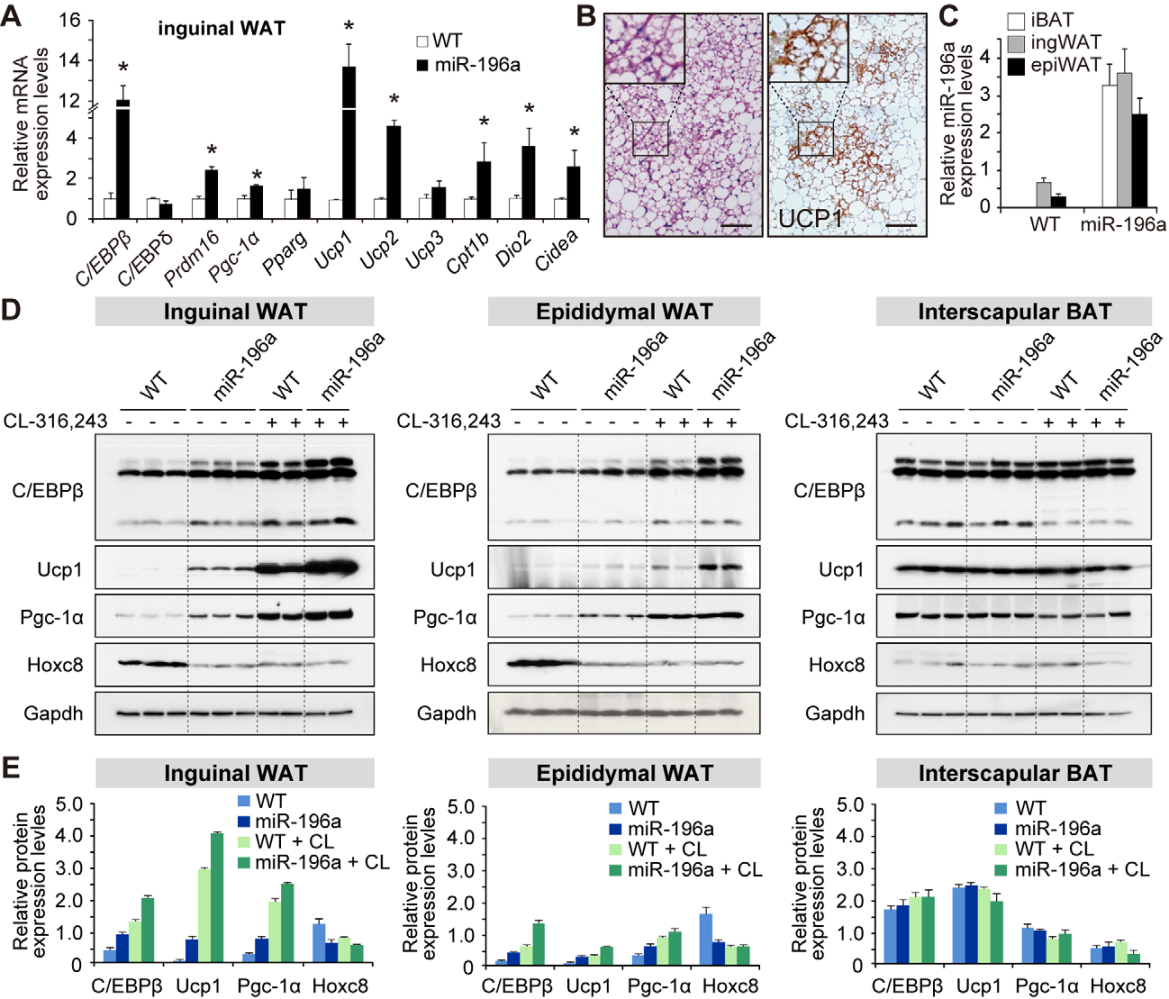
miR-196a induces brown adipocyte-like cells in WAT.

In vivo, the gene-expression analysis revealed an induction of brown-fat genes, including *C/EBPβ*, *Prdm16*, and *Ucp1* in ingWAT (Figure 5A), and the histological analysis revealed clusters of multilocular cells with Ucp1 expression (Figure 5B). It is known that different WAT depots respond to brown fat-inducing stimulations to different extents [31], and we therefore addressed the responses to the miR-196a expression in different fat depots. The miR-196a expression levels were comparable among the different fat depots in the miR-196a mice (Figures 5C and S10). The induction of C/EBPβ, Ucp1, and Pgc-1α was more prominent in the ingWAT than in the epiWAT (Figure 5D and 5E) and was further augmented after CL-316,243 treatment (Figure 5D and 5E). In the iBAT, no appreciable influence of miR-196a was observed (Figure 5D and 5E). Thus, miR-196a induces the brown adipocyte-like cells with characteristic appearance and gene expression profile of brown adipocytes in WAT.

### The miR-196a Mice Show Resistance to Obesity and Improved Glucose Metabolism

**Figure 6:**
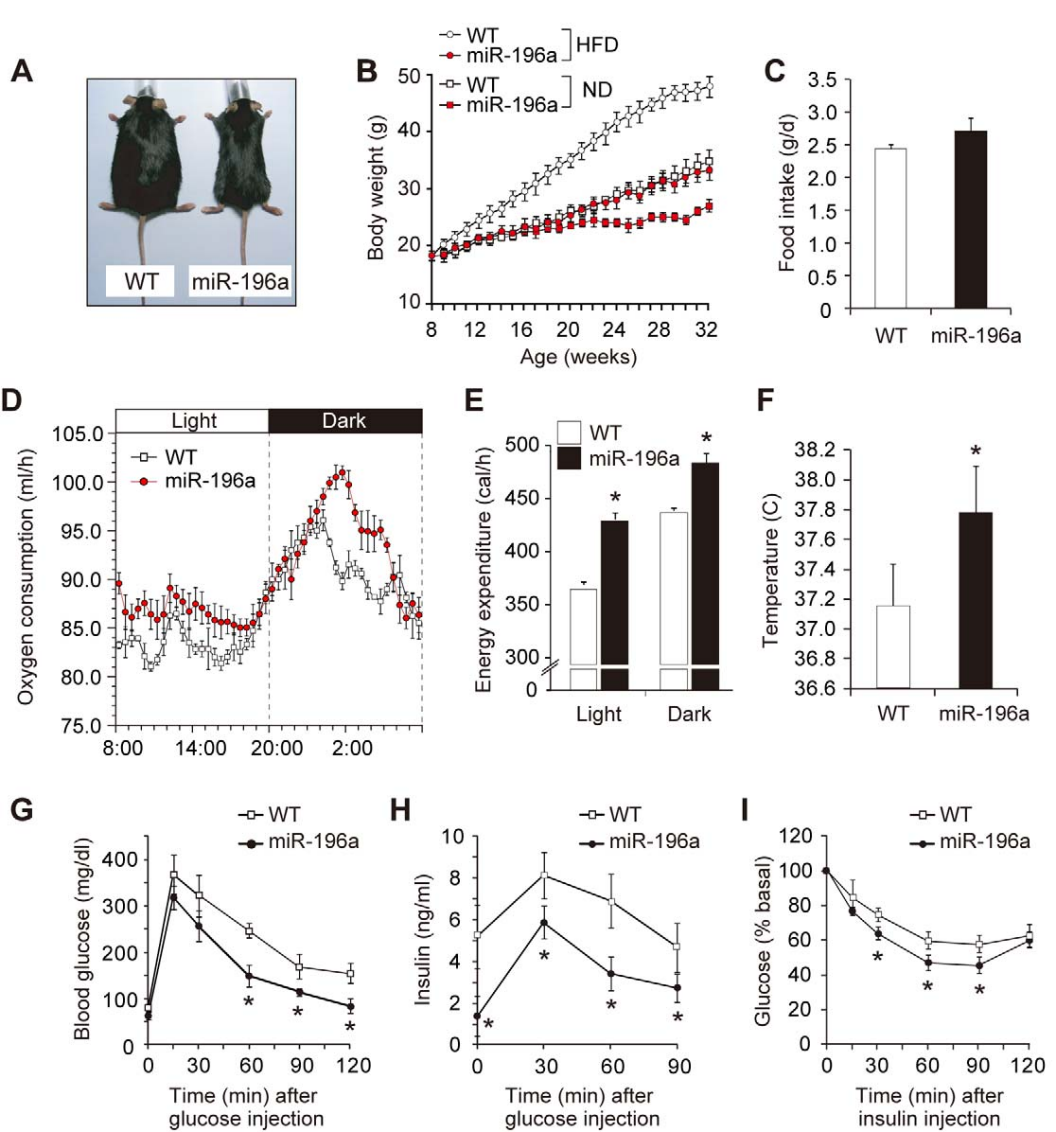
The miR-196a mice show resistance to obesity and improved glucose metabolism. (A) The appearance of the WT and miR-196a mice fed a high-fat diet for 16 wk. (B) The body weights of the WT and miR-196a mice (*n* = 8) fed a high-fat diet (HFD) or normal diet (ND) after 8 wk old. (C) The daily food intake of the WT and miR-196a mice (*n* = 8). (D) Oxygen consumption rates (V̇O2) in the WT and miR-196a mice fed a normal diet (*n* = 6). Measurements were performed on 3- to 4-mo-old mice with similar body weight that were given a standard diet. (E) The energy expenditure in the WT and miR-196a mice fed a normal diet (*n* = 6) calculated based on V̇O2 and V̇CO2 values and averaged separately for the light and dark phases. Measurements were performed on 3- to 4-mo–old mice with similar body weight that were given a standard diet. (F) The core body temperatures of the WT and miR-196a mice (*n* = 6). (G) The glucose tolerance test results for the WT and miR-196a mice (*n* = 10). (H) The plasma insulin concentrations after glucose injection in the WT (*n* = 8) and miR-196a (*n* = 10) mice. (I) The insulin tolerance test for the WT and miR-196a mice (*n* = 10). All data are presented as means ± SEM; * *p*<0.05.

Based on the finding that miR-196a is capable of inducing the brown adipocyte-like cells, we next addressed whether they were metabolically functional. The miR-196a mice showed a tendency to be leaner than WT mice (Figure 6B), and even when fed a high-fat diet, they exhibited resistance to obesity (Figure 6A and 6B), despite the fact that their food intake tended to be increased compared with that of the WT littermates (Figure 6C). The weight reduction was attributable to a reduced fat accumulation (Figure S11). To interrogate the mechanism behind the obesity resistance of the miR-196a mice, indirect calorimetry was performed. We used mice with similar body weight under a normal diet. As a result, the oxygen consumption (Figure 6D) and the energy expenditure (Figure 6E and Table S1) were enhanced during both the light and dark phases in the miR-196a mice compared to the WT mice, indicating the accelerated energy metabolism. The difference of the oxygen consumption and the energy expenditure was even enlarged when the mice were fed a high-fat diet (Figure S12). The core body temperature was higher in the miR-196a mice than in the WT mice (Figure 6F). These findings suggest that miR-196a boosted the cellular energy combustion through the induction of brown adipocyte-like cells. We next analyzed impacts of miR-196a on glucose metabolism in the miR-196a mice. In the glucose tolerance tests, the miR-196a mice showed lower blood glucose (Figure 6G) and insulin levels (Figure 6H). After insulin administration, they exhibited more pronounced declines in their blood glucose levels (Figure 6I). These results imply that miR-196a prevented the mice from developing insulin resistance, the premorbid condition of type 2 diabetes. Taken together, these findings suggest that the miR-196a-induced brown adipocyte-like cells are metabolically functional and have favorable impacts on glucose metabolism in mice.

### Hoxc8 Targets C/EBPβ in Cooperation With HDAC3 to Regulate Brown-Fat Genes

**Figure 7:**
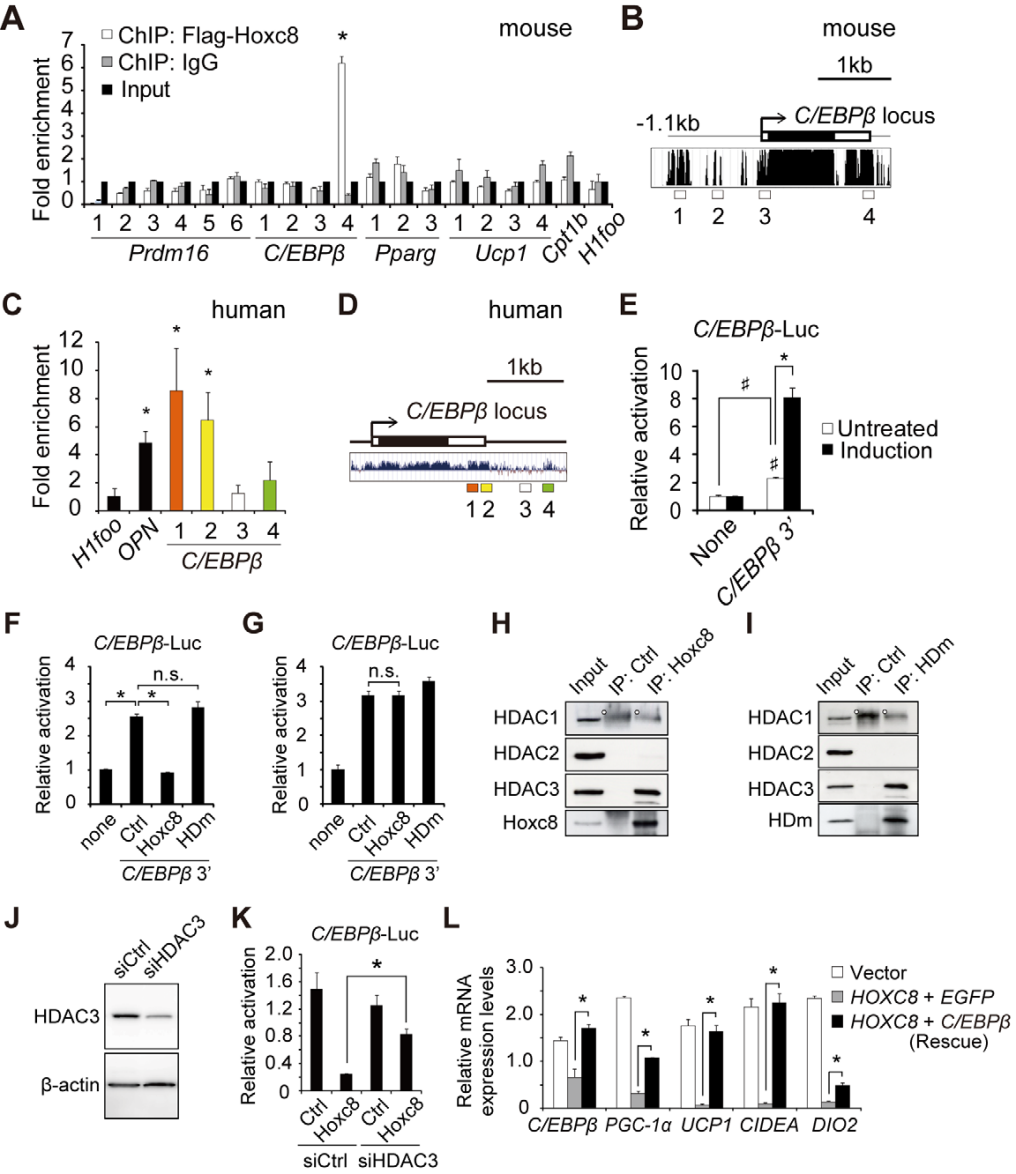
HOXC8 targets *C/EBPβ* in cooperation with HDAC3 to regulate brown-fat genes. (A) The ChIP analysis in 3T3-L1 preadipocytes expressing Flag-*Hoxc8* in mouse *C/EBPβ* locus. *H1foo* is an oocyte-specific gene and served as a negative control. The numbers 1–4 in *C/EBPβ* correspond to 1–4 in (B), respectively. (B) The interspecies conservation of the mouse *C/EBPβ* 3′. The data obtained from the UCSC Genome Browser map. (C) The ChIP analysis in the human WAT-progenitor cells in human *C/EBPβ* locus. *Osteopontin* (*OPN*) served as a positive control. (D) The interspecies conservation and location of ChIP primers used in (C) in the human *C/EBPβ* locus. The data obtained from the UCSC Genome Browser map. (E) Luciferase reporter assay to assess the transcriptional activity of *C/EBPβ* 3′ sequence inserted into the 3′ end of the luciferase gene. The activity was measured in 3T3-L1 preadipocytes left untreated (Untreated) or induced to undergo adipogenesis (induction). * *p*<0.05. ^♯^ *p*<0.05. (F) Luciferase reporter activity of *C/EBPβ* 3′ sequence measured in 3T3-L1 preadipocytes transfected with *Hoxc8*, homeodomain-mutated *Hoxc8* (HDm), or control vector. (G) Luciferase reporter activity in 3T3-L1 preadipocytes transfected with Hoxc8, HDm, or control vector in the presence of trichostatin A, a histone deacetylase (HDAC) inhibitor. (H) Immunoprecipitation in 3T3-L1 preadipocytes stably expressing Flag-Hoxc8. The immunoblot analysis was performed after immunoprecipitation with anti-Flag antibody. The white dot indicates a non-specific band. (I) Immunoprecipitation in 3T3-L1 preadipocytes stably expressing Flag-HDm. The immunoblot analysis was performed after immunoprecipitation with anti-Flag antibody. The white dot indicates a non-specific band. (J) The immunoblot of HDAC3 in the 3T3-L1 preadipocytes transfected with siRNA against *HDAC3*. (K) Luciferase reporter activity in 3T3-L1 preadipocytes transfected with *Hoxc8* and siRNA against *HDAC3*. (L) The mRNA expression levels in the human WAT-progenitor cells stably expressing *HOXC8* followed by transfection with *C/EBPβ* or *EGFP* and adipogenic induction. All data are presented as means ± SEM. * *p*<0.05. n.s., not significant.

The concept that miR-196a induces brown adipogenesis through the suppression of Hoxc8, which might function as a gatekeeper of brown adipogenesis in WAT, facilitated us to investigate the target gene of Hoxc8 transcription factor. The chromatin immunoprecipitation (ChIP) assays among the candidate genes revealed a significant enrichment of Hoxc8 in the *C/EBPβ* locus in the mouse genome (Figure 7A). *C/EBPβ* is a crucial regulator of brown adipogenesis, which is highly expressed in BAT compared to WAT [23]. The enrichment was found in the 3′ region, which harbors high interspecies conservation (Figure 7B, “4”). In human WAT-progenitor cells, too, the enrichment of HOXC8 was observed in the *C/EBPβ* 3′ region (Figure 7C and 7D). The enrichment of HOXC8 was also observed in the promoter of *osteopontin* (*OPN*) gene used as a positive control (Figure 7C) [32]. To ask whether the binding of Hoxc8 in the 3′ of *C/EBPβ* has a regulatory role, we performed the reporter assay by replacing the *C/EBPβ* coding region with *luciferase* gene. Indeed, the *C/EBPβ* 3′ sequence induced luciferase activity, which was further augmented by adipogenic stimulation (Figure 7E). This luciferase expression was suppressed by concomitant transfection of *Hoxc8* but not by that of *Hoxc8* with a mutated homeodomain (HDm) lacking DNA-binding capacity (Figure 7F) [33]. These results implied that Hoxc8 regulates the *C/EBPβ* expression via the *C/EBPβ* 3′ regulatory sequence. Furthermore, the suppressive effect of Hoxc8 was abolished by trichostatin A, a histone deacetylase (HDAC) inhibitor, indicating that the suppressive effect involves histone deacetylation (Figure 7G). In this regard, Hoxc8 interacted with HDAC3 (Figure 7H) [34]–[35], but not with HDAC1 or HDAC2. The interaction was independent of the DNA binding capacity of Hoxc8 (Figure 7I). To further corroborate that *HDAC3* cooperates with *Hoxc8*, *HDAC3* was suppressed using siRNA (Figure 7J), resulting in partial elimination of the suppressive effects of *Hoxc8* (Figure 7K). To demonstrate that *C/EBPβ* is an essential target of Hoxc8, *C/EBPβ* was transfected into the human WAT-progenitor cells that stably expressed human *HOXC8*, resulting in restoration of the brown-fat gene expression that had been suppressed by *HOXC8* (Figure 7L). Thus, Hoxc8 targets and represses *C/EBPβ* in an HDAC3-dependent manner.

In summary, during the brown adipogenesis induced by cold exposure or β3-adrenergic stimulations, miR-196a is induced in WAT-progenitor cells and suppresses Hoxc8, which targets *C/EBPβ*, an essential regulator of brown adipogenesis. The miR-196a expression is required for the brown-fat gene expression and sufficient to induce metabolically functional brown adipocyte-like cells in WAT in mice. Our findings imply the therapeutic potential of targeting the miR-196a-*Hoxc8*-*C/EBPβ* signaling pathway that induces metabolically functional brown adipocytes in WAT to treat obesity and its associated diseases.

## Discussion

**Figure 8:**
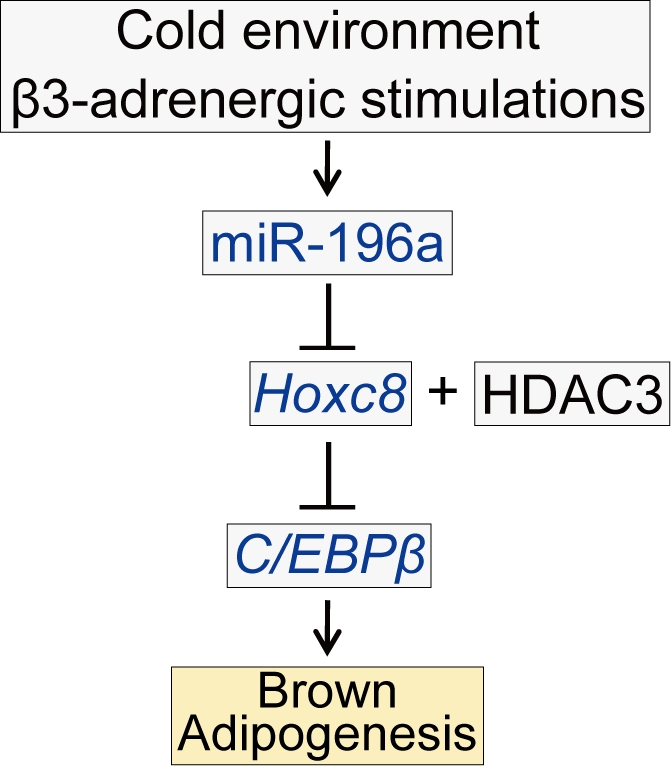
A schematic of miR-196a-regulated brown adipogenesis of WAT-progenitor cells. Cold temperatures or β3-adrenergic stimulations induce miR-196a in the WAT-resident progenitor cells in mice. miR-196a post-transcriptionally suppress *Hoxc8*, which is one of the white-fat genes. The direct target of Hoxc8 is *C/EBPβ*, a master switch of brown adipogenesis that provokes brown fat gene program in the WAT-progenitor cells.

Recent discoveries of metabolically active BAT in adult humans have highlighted BAT as a therapeutic target for treating obesity and its associated diseases. The brown adipocyte-like cells in WAT can be generated by cold exposure or β-adrenergic stimulation in rodents, but the molecular mechanisms underlying these phenomena have not been fully elucidated. In this work, we elucidated that miR-196a induces functional brown adipocytes in WAT in mice. miR-196a is upregulated in WAT-progenitor cells during brown adipogenesis induced by cold or β-adrenergic stimulations. miR-196a is required for the brown fat gene expression and is sufficient to induce metabolically functional brown adipocyte-like cells in mice. The target gene of miR-196a is *Hoxc8*, which is categorized as a white-fat gene with a previously undermined role in adipogenesis. Hoxc8 directly targets and represses *C/EBPβ*, a master switch of brown adipogenesis. Thus, the miR-196a-*Hoxc8*-*C/EBPβ* pathway underlies the brown adipogenesis in WAT (Figure 8) and might be a therapeutic target for the treatment of obesity and type 2 diabetes.

Elucidation of the molecular mechanism regulating the brown adipogenesis in WAT is important from both a biological and clinical viewpoint. Recent studies uncovered the existence of WAT-progenitor cells that harbor a potential to differentiate to brown adipocytes [16],[21]–[22],[36]. The molecular mechanism behind the inducible brown adipogenesis in WAT is relatively unknown, but recent studies elucidated the importance of *cyclooxygenase-2* [36]–[37] and *Prdm16* [38]. *C/EBPβ* is an essential regulator of brown fat gene program [23],[39]–[41], but whether *C/EBPβ* has a significant role in the inducible brown adipogenesis was not fully understood. We found that miR-196a suppresses *Hoxc8*, thereby derepressing *C/EBPβ*, which leads to the activation of the brown fat gene program. Our findings imply the relevance of *C/EBPβ* not only in the conventional brown adipogenesis but also in the inducible brown adipogenesis in WAT.

The cellular origin of the inducible brown adipocyte-like cells in WAT is an important question. Transdifferentiation is a significant mechanism that has been reported to contribute to brown adipocyte recruitment in WAT [42]–[43]. Because the increase in *Ucp1* mRNA is detectable within a few hours after cold stimulation [1],[31], and in vitro SVF cell differentiation is a longer process, transdifferentiation might have a significant role in the rapid response to stimulation. The important questions include the relative contribution of transdifferentiation and the progenitor cell-mediated mechanism in brown adipocyte recruitment throughout the different phases upon exposure to a cold environment and physiological energy regulation.

miRNAs regulate the gene networks underlying various physiological and pathological phenomena and might be therapeutic targets [18]–[19],[44]–[46]. miR-196a has been implicated in the in vitro osteoblast differentiation of human fat progenitor cells, where miR-196a suppresses Hoxc8 [47], but the in vivo relevance remains unknown. We elucidated that miR-196a is induced in the WAT-progenitor cells after the induction of brown adipogenesis, is required for the induction of brown fat gene expression, and is sufficient to induce the metabolically functional brown adipocyte-like cells in WAT.

Our observations indicate that miR-196a has only a modest, if any, effect on iBAT. The endogenous expression of Hoxc8 and miR-196a was much lower in iBAT than in ingWAT and epiWAT. The forced expression of miR-196a in mice did not yield appreciable effects in iBAT. Treatment of mice with β3-adrenergic receptor agonists usually leads to a much more moderate induction of *Ucp1* expression in iBAT than in WAT depots. Although the primary cultures of brown adipocytes from iBAT are highly sensitive to β3-adrenergic activation [1], a moderate but significant induction of *Ucp1* was reported in iBAT in response to β3-adrenoreceptor agonists in vivo [48]. A relatively modest response from iBAT to the β3-adrenergic receptor agonist compared with subcutaneous and visceral WAT has also been reported in other studies [16],[43],[49]. These results imply that distinct machinery regulates brown adipocyte recruitment in iBAT, which was previously suggested by Petrovic et al. [21].

A number of miRNAs function as a molecular switch [46],[50]–[53], and further elucidating how the miRNAs influence the physiological output will enable better understanding and clinical use of miRNAs.

The significance of the distinct expression patterns of Hox genes between BAT and WAT has been unknown [10]–[12]. We here demonstrate that Hoxc8 functions as an important determinant of white fat lineage and negatively regulates the induction of brown adipogenesis in WAT-progenitor cells by repressing *C/EBPβ*, which is a master switch of brown adipogenesis [39]–[41]. Mechanistically, Hoxc8 directly represses the *C/EBPβ* expression through the 3′ regulatory sequence. Similar conserved non-coding regulatory elements have been reported for the *Foxp3* gene [54], and previous studies suggested that the majority of transcription factors bind to sites other than the promoter [20],[55]. Hoxc8 recruits HDAC3, which is implicated in the regulation of metabolic genes [34],[35]. Since the HDAC proteins lack DNA-binding activity, they are recruited to target genes via association with transcriptional factors [56]. Our findings imply the possible therapeutic efficacy of HDAC inhibitors for obesity through inducing brown adipogenesis, but further study is required to address the possibility.

The induction of brown adipogenesis in WAT has great therapeutic potential. Our findings suggest that the miR-196a-*Hoxc8*-*C/EBPβ* pathway may constitute a promising strategy for addressing the social and health problems caused by obesity and its associated diseases.

## Materials and Methods

### Ethics Statement

Mice were handled in accordance with protocols approved by the Ethics Committee for Animal Experiments of the Osaka University Graduate School of Medicine.

### Plasmids

The coding sequence of human *Hoxc8* (Gene ID: 3224) was cloned into pCX4-puro [57] and pCAGIP vector [58]. The pCX4-*Hoxc8* retroviral vector was used to generate human WAT-progenitor cells stably expressing *Hoxc8*. Human *C/EBPβ* was cloned into the pCAGIP vector. The homeodomain mutant (I195A/Q198A/N199A/M202A) [33] of Hoxc8 (HDm) was created by site-directed mutagenesis. For lentivirus-mediated shRNA expression, pLenti6-miR-196a, -shHoxc8, and -shLacZ were generated from pcDNA6.2 constructs by Gateway reactions. Lentivirus was generated by cotransfection of the pLenti6 construct with packaging plasmids into 293FT cells according to the manufacturer's instruction (Invitrogen). For Hoxc8 3′UTR analysis, human Hoxc8 3′UTR sequence was cloned and inserted to the 3′ end of Hoxc8 cDNA. The miR-196a binding site (CCCAACAACTGAGACTGCCTA) was deleted to generate Hoxc8-ΔmiR-196a-BS.

### Gene Expression Analysis

Total RNA was isolated using the RNeasy Lipid Tissue Mini Kit (QIAGEN, CA). Reverse transcription and quantitative PCR were performed as previously described [59]. For microRNA quantification, total RNA was isolated using a mirVana miRNA isolation kit (Applied Biosystems). Reverse transcription and quantitative PCR were performed according to the manufacturer's instructions. A list of probes is provided in Text S1.

### RNA-seq

RNA from human white fat (WAT) progenitor cells was extracted with RNeasy (QIAGEN) following the manufacturer's instructions. 12.5 µg of total RNA were subjected to two rounds of oligo-dT purification using Ambion MicroPoly(A) Purist Kit (Ambion). 50 ng of the fragmented poly(A) RNA by using RNaseIII were ligated to SOLiD Adaptor Mix and were reverse-transcribed by using SOLiD Total RNA-Seq Kit (Life Technologies). First-strand cDNA from 100 bp to 150 bp was selected by using Agencourt AMPure XP reagent (Beckman Coulter Genomics) and was amplified by SOLiD 5′ PCR primer and barcoded SOLiD 3′ PCR primers (Life Technologies). Sequencing libraries were prepared according to Life Technologies' protocol. RNA-seq libraries were sequenced with SOLiD 4. Mapping of resulting reads was performed by Bioscope (Life Technologies), and analysis of mapped reads (31,825,850 reads in hADSC_1 and 42,009,231 reads in hADSC_2) was performed by Cufflinks [60].

### Cell Culture

Human WAT-progenitor cells were isolated from human flank subcutaneous fat lipoaspirate (Lonza, Switzerland) and maintained in mesenchymal stem cell growth medium (Lonza). For adipogenesis, 2-d post-confluent cells were treated with an induction medium containing 0.5 mM IBMX, 10 µg/ml insulin, and 1 µM dexamethasone (MDI). The induction medium was changed every 2 d. Forskolin (40 µM, Sigma-Aldrich) was added to the medium as noted. Antisense oligonucleotide against miR-196a (Anti-miR miRNA inhibitor, AM10068, Ambion) was transfected according to the manufacturer's instruction. The fat progenitor cells were isolated from inguinal white adipose tissue (WAT) or interscapular BAT (iBAT) of C57Bl/6 mice using a standard method [61]. Adipogenic induction was performed by treating the cells with the induction medium for 2 d.

### Western Blot Analysis

Western blotting was performed with antibodies against Hoxc8 (1∶1,000, ab86236, abcam), C*/EBPβ* (1∶200, sc-150, Santa Cruz Biotechnology, CA), UCP1 (1∶1,000, U6382, Sigma-Aldrich), PGC-1α (1∶1,000, ab54481, abcam), β-actin (1∶5,000, AC-15, Sigma-Aldrich), and GAPDH (1∶5,000, ab8245, abcam). The secondary antibodies (GE Healthcare) were used at a 1∶1,000 dilution ratio. Immunoreactive bands were detected with Chemi-LumiOne L (Nacalai Tesque) or ECL plus (GE Healthcare). Densitometry was performed with the ImageJ software (NIH; http://rsb.info.nig.gov/ij/).

### Immunocytochemistry

Immunocytochemistry was performed using antibodies against Hoxc8 (1∶200, MMS-286R, Covance), Hoxc6 (1∶200, ab41587, Abcam), Pgc-1α (1∶300, ab54481, Abcam), or UCP1 (1∶500, ab10983, Abcam) as previously described [59]. The primary antibodies were detected using anti-mouse-Alexa Fluor 546, anti-mouse-Alexa Fluor 488, or anti-rabbit-Alexa Fluor 546 (1∶1,000, Invitrogen). Cells were counterstained with CellTracker Green Bodipy (Invitrogen), Bodipy 493/503 (D3922, Invitrogen), and 4′-6-diamidino-2-phenylindole (DAPI, Invitrogen).

### Mice

These experiments were approved by the Ethics Committee for Animal Experiments of the Osaka University Graduate School of Medicine. Male outbred C57Bl/6 mice were used. For acute cold-exposure studies, 3- to 4-mo-old male mice were housed at 4°C for 5 h. For β3-adrenaline receptor stimulation, CL-316,243 (Sigma), at 0.5 mg/kg, was injected intraperitoneally once daily for 7 d. Transgenic mice with fat-specific forced expression of miR-196a were generated using a transgene encoding miR-196a driven by the enhancer/promoter of the *aP2* gene [30], and littermates were used as the wild-type controls.

### Histological Analysis

Inguinal fat sections were fixed in 10% buffered formalin and stained with hematoxylin-eosin. For immunohistochemistry, paraffin-embedded sections were incubated with antibodies against UCP1 (1∶1,000, ab10983, Abcam) followed by detection using ABC Vectastain-Elite kit (Vector Labs). Nuclei were counterstained with modified Mayer's hematoxylin (Diagnostic BioSystems).

### miRNA In Situ Hybridization

Inguinal WAT depots of mice were dissected after perfusion and fixation with Tissue Fixative (Genostaff), embedded in paraffin, and sectioned at 6 µm. The sections were de-waxed with xylene and rehydrated. The sections were fixed with 4% paraformaldehyde (PFA) for 15 min, treated with 8 µg/ml proteinase K for 30 min at 37°C, re-fixed with 4% PFA, and placed in 0.2 N HCl for 10 min. The sections were acetylated with 0.1 M tri-ethanolamine-HCl, pH 8.0, and 0.25% acetic anhydride for 10 min. After being washed with PBS, the sections were treated with PBS at 80°C for 5 min. The sections were hybridized with 3′-digoxygenated probes (18 pmol/ml, miR-196a-AS-LNA1: cCcaAcaAcaTgaAacTacCta, Control (Ctrl)-LNA1: cGacTacAcaAatCagCgaTtt, capitals denote LNA) in Probe Diluent-1 (Genostaff) at 50°C for 16 h and washed in 5× HybriWash (Genostaff) at 50°C for 20 min, 50% formamide in 2× HybriWash at 50°C for 20 min, twice in 2× HybriWash at 50°C for 20 min, and twice in 0.2× HybriWash at 50°C for 20 min. The sections were treated with 0.5% blocking reagent (Roche) in TBST for 30 min and incubated with anti-DIG AP conjugate (1∶1,000, Roche) for 2 h at RT. The sections were washed twice with TBST and incubated in a solution with a composition of 1,000 mM NaCl, 50 mM MgCl_2_, 0.1% Tween-20, 100 mM Tris-HCl, pH 9.5. Coloring reactions were performed with NBT/BCIP solution (Sigma) overnight followed by counterstaining with Kernechtrot stain solution (Mutoh).

### Metabolic Measurements

Mice were given a standard diet or a high-fat diet (20.4% protein, 33.2% fat, 46.4% carbohydrates by calories; MF+; Oriental Yeast Co., Japan). Metabolic measurements were performed on 3- to 4-mo-old mice with similar body weight that were given a standard diet. Food intake and body weight were measured daily and weekly, respectively. For glucose tolerance tests, the mice were deprived of food for 16 h and were injected intraperitoneally with glucose (2 g/kg). For insulin tolerance tests, the mice were allowed *ad libitum* access to food followed by an intraperitoneal injection of human insulin (0.75 U/kg, Eli Lilly). The plasma concentration of glucose was measured with a Glucometer (Sanwa Kagaku Kenkyusho, Japan), and insulin was measured with an ELISA (Morinaga Institute of Biological Science, Japan). Indirect calorimetry was performed under 12 h light and dark cycles beginning at 8:00 a.m. and 8:00 p.m., respectively. After 1 d of acclimation, V̇O_2_ and V̇CO_2_ were recorded every 3 min over 3 d using the Metabolism Measurement System (MK-5000, Muromachi Kikai, Japan). Energy expenditure (EE) was calculated using the equation of Weir: EE (kcal/kg/h) = (3.815×V̇O_2_)+(1.232×V̇CO_2_). For body temperature measurement, mice were housed singly and unrestrained and had free access to food and water. Body temperature was measured using a rectal probe (Perimed, Sweden).

### Native ChIP Assays

Chromatin immunoprecipitation was performed as previously described [62] with 3T3-L1 preadipocytes expressing Flag-tagged human Hoxc8. Primer sequences are listed in Text S1.

### Luciferase Assays

The *C/EBPβ3′*-luciferase constructs (*C/EBPβ*-Luc) were generated by cloning the 3′ sequence of the human *C/EBPβ* gene (+1,021 to +1,837) into the downstream of luciferase gene in pGL3 promoter plasmid (Promega). Dual luciferase assays were performed as previously described [62] with 3T3-L1 preadipocytes. Trichostatin A (330 nM, Sigma-Aldrich) was added 4 h after transfection as indicated. Mission siRNA (Sigma) for HDAC3 (sense: 5′GUAUCCUGGAGCUGCUUAATT, antisense: 5′UUAAGCAGCUCCAGGAUACTT) was transfected using Neon transfection system (Invitrogen).

### Immunoprecipitation Analysis

Nuclear extracts were prepared as previously described [62] from 3T3-L1 preadipocytes transfected with Flag-*Hoxc8*, pretreated with Protein G Sepharose beads (Amersham Bioscience), and incubated with anti-Flag M2 Affinity Gel (A2220, Sigma-Aldrich) or control mouse IgG AC (Santa Cruz) overnight at 4°C. The beads were washed 3 times with nuclear isolation buffer containing 500 mM NaCl and 0.15% NP-40. Purified proteins were subjected to immunoblotting using antibodies against HDAC1 (3∶1,000, Millipore), HDAC2 (1∶2,000, H3159, Sigma), and HDAC3 (1∶500, ab16047, Abcam).

### Statistics

The statistical analysis was performed with StatView 5.0 software, JMP8 (SAS Institute, NC) and SPSS (IBM). All results are expressed as mean ± SEM. The data were compared using ANOVA, followed by Dunnett's test for pairwise comparisons against controls and by Tukey's test for multiple comparisons. For the analysis of energy expenditure, a one-way analysis of covariance (ANCOVA) was conducted. The body weight was used as the covariate. Statistical significance was defined as *p*<0.05.

### Accession Numbers

The RNA-seq data have been submitted to the NCBI Sequence Read Archive (SRA). The accession number is SRA048274.1.

## Supporting Information

Figure S1 The gene-expression analysis in human WAT-progenitor cells. (A) The summary of the microarray results from human WAT-progenitor cells transduced with Hoxc8 or control vector followed by adipogenic induction for 14 d. The expression levels were compared to those in the untreated cells and the fold changes in the expression levels are shown. (B) The immunofluorescence analysis of HOXC8 and PGC-1α in human WAT-progenitor cells induced to undergo differentiation for 14 d. The nuclei are stained with DAPI. The scale bar indicates 100 µm. (C) The immunofluorescence analysis of HOXC6 in human fat progenitor cells left untreated (Undifferentiated) or induced to undergo differentiation for 14 d (Adipogenic induction). The HOXC6 expression is maintained in the differentiated cells (arrowheads) that exhibit multiple vesicles. The nuclei are stained with DAPI. DIC, differential interference contrast. The scale bar indicates 50 µm. (D) The tissue distribution of HOXC6 expression in mice. The data are normalized to 18S. All data are presented as means ± SEM.
(TIF)

Figure S2 Hoxc8 expression in mouse tissues. Hoxc8 expression is higher in white adipose tissue (WAT) than in brown adipose tissue (BAT) and other tissues. The real-time PCR results are normalized to β-actin.
(EPS)

Figure S3 The expression of miR-196a and Hoxc8 in SVF and adipocyte fraction. (A) miR-196a expression levels in SVF and adipocyte fraction from mice treated with CL-316,243 or saline. The results are normalized to U6. (B) Western blot analysis of Hoxc8 in SVF and adipocyte fraction from mice treated with CL-316,243 or saline.
(EPS)

Figure S4 The expression analysis for miR-196a. (A) miR-196a expression is upregulated during differentiation in the human WAT-progenitor cells. The results are normalized to U6. (B) The miR-196a expression is upregulated by treatment with forskolin in human WAT-progenitor cells. The results are normalized to U6. (C) The miR-196a expression levels in different tissues of the wild-type mice. The results are normalized to U6. (D) The miR-196a expression is not altered significantly during the differentiation of iBAT-derived SVF cells (conventional brown adipogenesis). The results are normalized to U6. All data are presented as means ± SEM. * *p*<0.05.
(EPS)

Figure S5 The expression analysis of Hoxc8 in iBAT-derived SVF cells. (A) Immunoblots of Hoxc8 in iBAT-SVF cells treated with or without adipogenic induction cocktail. The results of WAT-SVF cells were shown for comparison. β-actin served as a loading control. (B) Immunofluorescence analysis of Hoxc8 in the undifferentiated iBAT-SVF cells. The nuclei are stained with DAPI. The scale bar indicates 50 µm.
(TIF)

Figure S6 The analysis of human HOXC8 3′UTR. (A) The scheme of constructs. Human HOXC8 3′UTR sequence was inserted to the 3′ end of HOXC8 cDNA to generate pCX4-HOXC8-wild-type (wt) 3′UTR. The miR-196a complementary site was deleted to generate pCX4-HOXC8-ΔmiR-196-BS (binding site). (B) Immunoblots of HOXC8 in human WAT-SVF cells transduced with retroviral vector-encoded HOXC8, HOXC8-wt3′UTR, HOXC8-ΔmiR-196-BS, or control EGFP. The transduced cells were treated with or without adipogenic induction cocktail. β-actin served as a loading control. (C) The qRT-PCR analysis of brown-fat genes in the transduced cells induced to undergo differentiation for 14 d. The results are normalized to 18S. All data are presented as means ± SEM; * *p*<0.05.
(EPS)

Figure S7 The effects of Hoxc8 knockdown on the expression of brown fat genes. (A) The qRT-PCR analysis of adipogenesis-related genes in mouse WAT-SVF cells transduced with control shRNA or shRNA against Hoxc8 followed by adipogenic induction. The results were normalized to β-actin. The data are presented as means ± SEM; ** *p*<0.01. (B) Immunoblots in mouse SVF cells transduced with control shRNA, shRNA against Hoxc8, or miR-196a encoded by lentiviral vectors. β-actin served as a loading control.
(TIF)

Figure S8 Gene expression analysis in the WAT-progenitor cells derived from the miR-196a mice. (A) The fluorescent microscopic view of the SVF cells derived from the aP2-miR-196a mice maintained without adipogenic induction. The scale bar indicates 100 µm. (B) The miR-196a expression levels in the WAT-progenitor cells derived from inguinal WAT of the WT and miR-196a mice. Data were normalized to U6. (C,D) The Western blot (C) and immunofluorescence (D) analysis of Hoxc8 in the WAT-progenitor cells. The scale bars indicate 30 µm. (E) A confocal 3-D image of an inguinal WAT from a miR-196a mouse. The vasculature and nuclei were visualized using anti-CD31 antibody and DAPI, respectively. V, vasculature; F, fat cells. (F) The gene expression analysis in WAT-progenitor cells induced to undergo adipogenesis for 14 d. Data are presented as the mean ± SEM. * *p*<0.05, ** *p*<0.01 versus WT.
(TIF)

Figure S9 miR-196a functions in a cell-autonomous manner. (A) The qRT-PCR analysis of miR-196a in human WAT-SVF cells transduced with lentiviral vector-encoded miR-196a. The results are normalized to U6. (B) Immunoblots of HOXC8 in human WAT-SVF cells transduced with miR-196a or control miR-LacZ. β-actin served as a loading control. (C) The qRT-PCR analysis of brown fat genes in human WAT-SVF cells transduced with miR-196a or control miR-LacZ followed by adipogenic induction. All data are presented as means ± SEM. * *p*<0.05.
(TIF)

Figure S10 The miR-196a expression levels in tissues of the miR-196a mice. The qRT-PCR analysis of miR-196a in tissues of the miR-196a mice. The results are normalized to U6. All data are presented as means ± SEM.
(EPS)

Figure S11 The weight reduction in the miR-196a mice is attributable to a reduced fat accumulation. (A) Body length does not differ significantly between the WT and miR-196a mice (*n* = 6). (B) The organ weights for the WT and miR-196a mice fed a high-fat diet for 16 wk (*n* = 3). The weight of the inguinal fat, epididymal WAT and liver is significantly lower in the miR-196a mice than in the WT mice. The WT mice exhibit more severe fatty livers than the miR-196a mice. All data are presented as means ± SEM. * *p*<0.05. (C) The appearance of the organs from the WT and miR-196a mice fed a high-fat diet for 16 wk.
(TIF)

Figure S12 Oxygen consumption rates and energy expenditure in the WT and miR-196a mice fed a high-fat diet. (A) Oxygen consumption rates (V̇O2) in the WT and miR-196a mice (*n* = 6) under a high-fat diet. (B) The energy expenditure in the WT and miR-196a mice (*n* = 6) under a high-fat diet calculated based on V̇O2 and V̇CO2 values and averaged separately for the light and dark phases (*n* = 6).
(EPS)

Table S1 ANCOVA analysis for energy expenditure by body weight.
(EPS)

Text S1 TaqMan probes and ChIP primers.
(PDF)

## Abbreviations

ASO: antisense oligonucleotide
BAT: brown adipose tissue
epiWAT: epididymal adipose tissue
iBAT: interscapular brown adipose tissue
ingWAT: inguinal white adipose tissue
SVF: stromal vascular fraction
WAT: white adipose tissue
