# Supplementary figures and images for: Essential Role for miR-196a in Brown Adipogenesis of White Fat Progenitor Cells
Source: PLoS Biol. 2012 Apr 24;10(4):e1001314. doi: 10.1371/journal.pbio.1001314 (PMC3335871; doi:10.1371/journal.pbio.1001314)

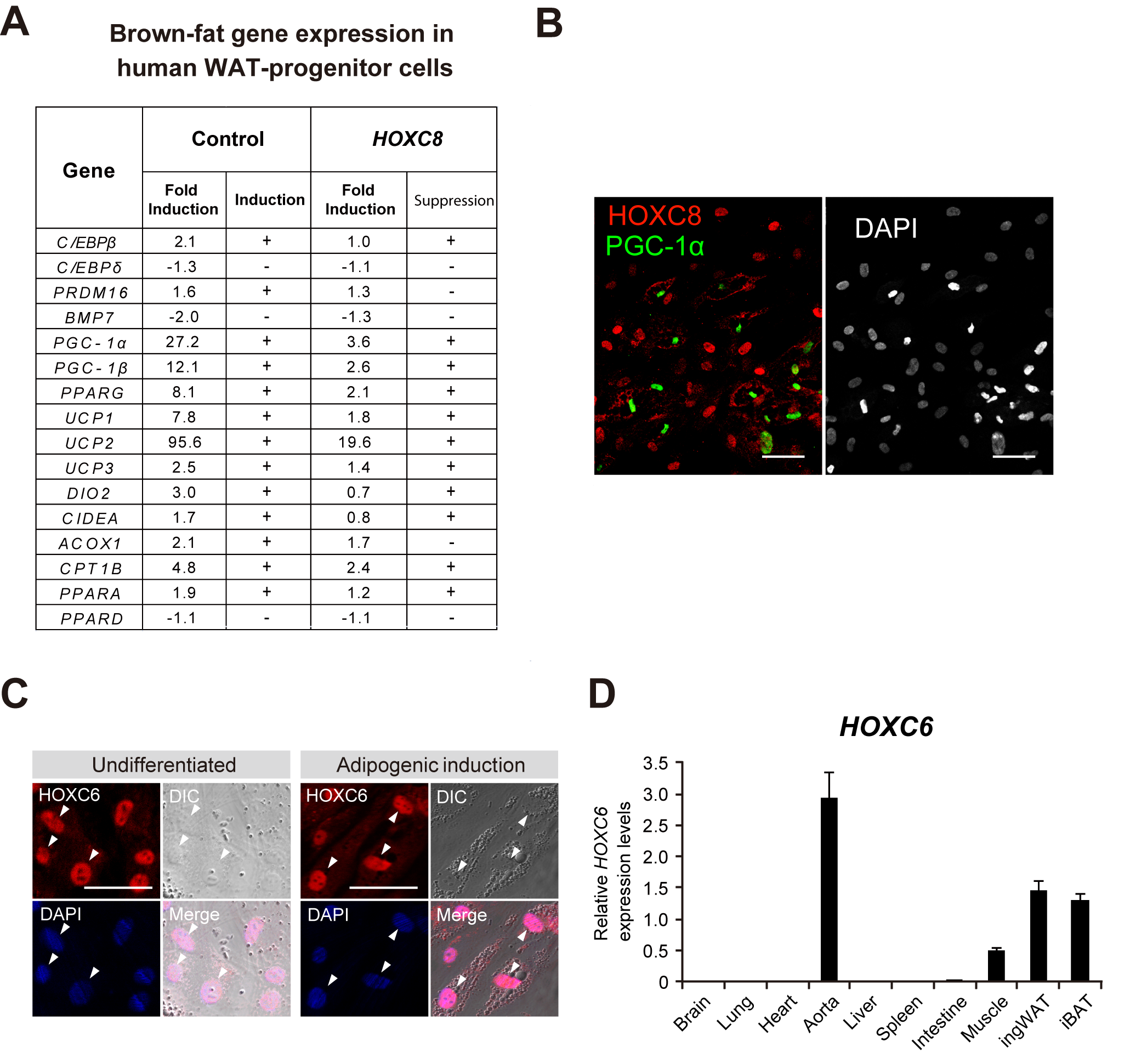

Supplement: Figure S1 — The gene-expression analysis in human WAT-progenitor cells. (A) The summary of the microarray results from human WAT-progenitor cells transduced with Hoxc8 or control vector followed by adipogenic induction for 14 d. The expression levels were compared to those in the untreated cells and the fold changes in the expression levels are shown. (B) The immunofluorescence analysis of HOXC8 and PGC-1α in human WAT-progenitor cells induced to undergo differentiation for 14 d. The nuclei are stained with DAPI. The scale bar indicates 100 µm. (C) The immunofluorescence analysis of HOXC6 in human fat progenitor cells left untreated (Undifferentiated) or induced to undergo differentiation for 14 d (Adipogenic induction). The HOXC6 expression is maintained in the differentiated cells (arrowheads) that exhibit multiple vesicles. The nuclei are stained with DAPI. DIC, differential interference contrast. The scale bar indicates 50 µm. (D) The tissue distribution of HOXC6 expression in mice. The data are normalized to 18S. All data are presented as means ± SEM. (TIF) [file pbio.1001314.s001.tif]

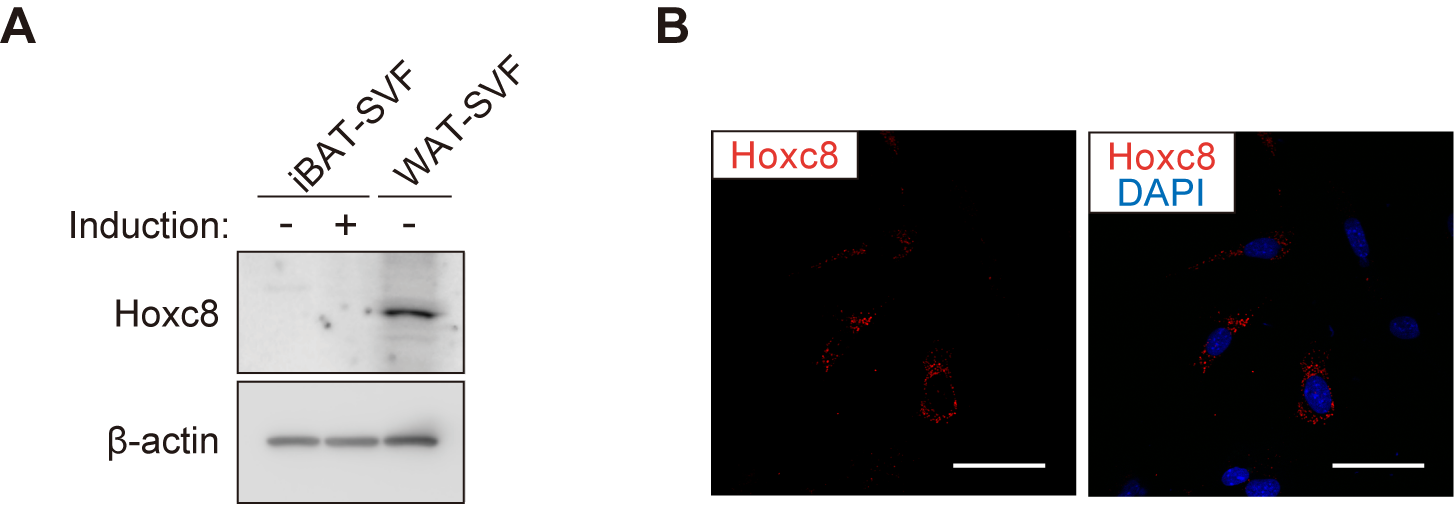

Supplement: Figure S5 — The expression analysis of Hoxc8 in iBAT-derived SVF cells. (A) Immunoblots of Hoxc8 in iBAT-SVF cells treated with or without adipogenic induction cocktail. The results of WAT-SVF cells were shown for comparison. β-actin served as a loading control. (B) Immunofluorescence analysis of Hoxc8 in the undifferentiated iBAT-SVF cells. The nuclei are stained with DAPI. The scale bar indicates 50 µm. (TIF) [file pbio.1001314.s005.tif]

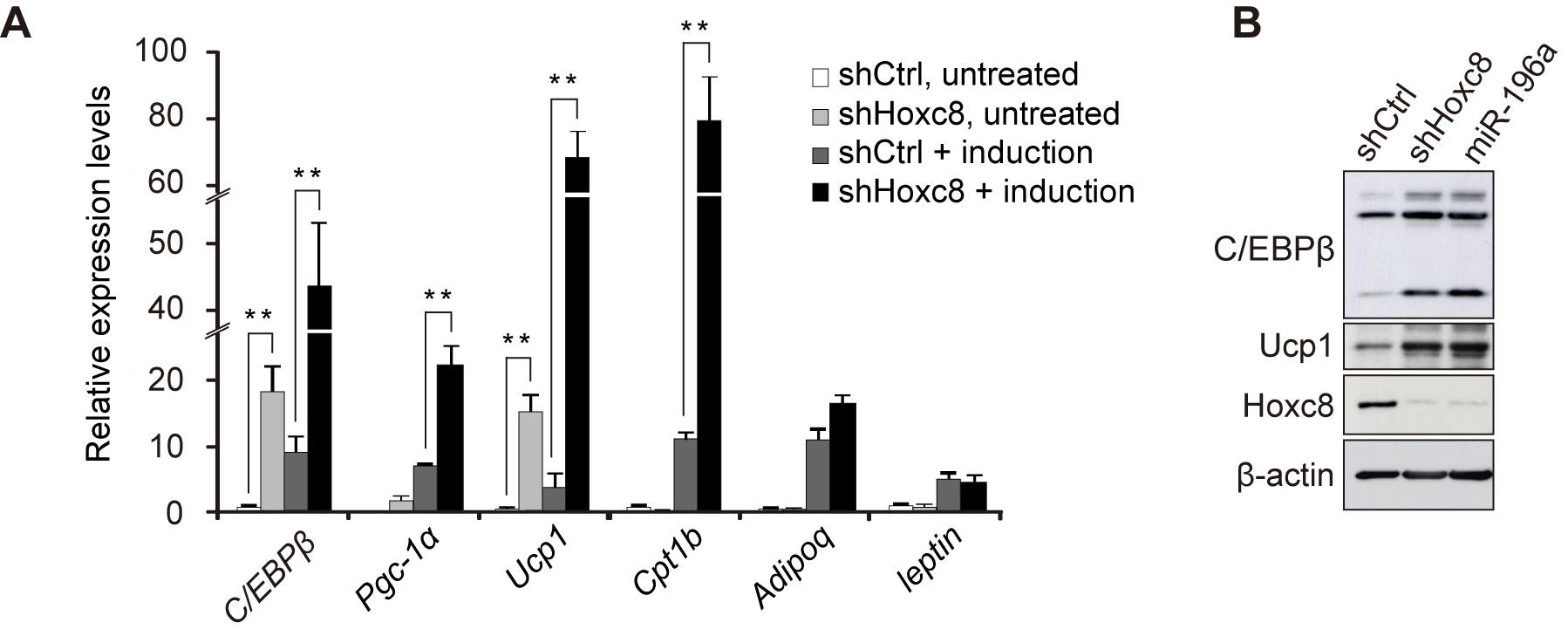

Supplement: Figure S7 — The effects of Hoxc8 knockdown on the expression of brown fat genes. (A) The qRT-PCR analysis of adipogenesis-related genes in mouse WAT-SVF cells transduced with control shRNA or shRNA against Hoxc8 followed by adipogenic induction. The results were normalized to β-actin. The data are presented as means ± SEM; ** p<0.01. (B) Immunoblots in mouse SVF cells transduced with control shRNA, shRNA against Hoxc8, or miR-196a encoded by lentiviral vectors. β-actin served as a loading control. (TIF) [file pbio.1001314.s007.tif]

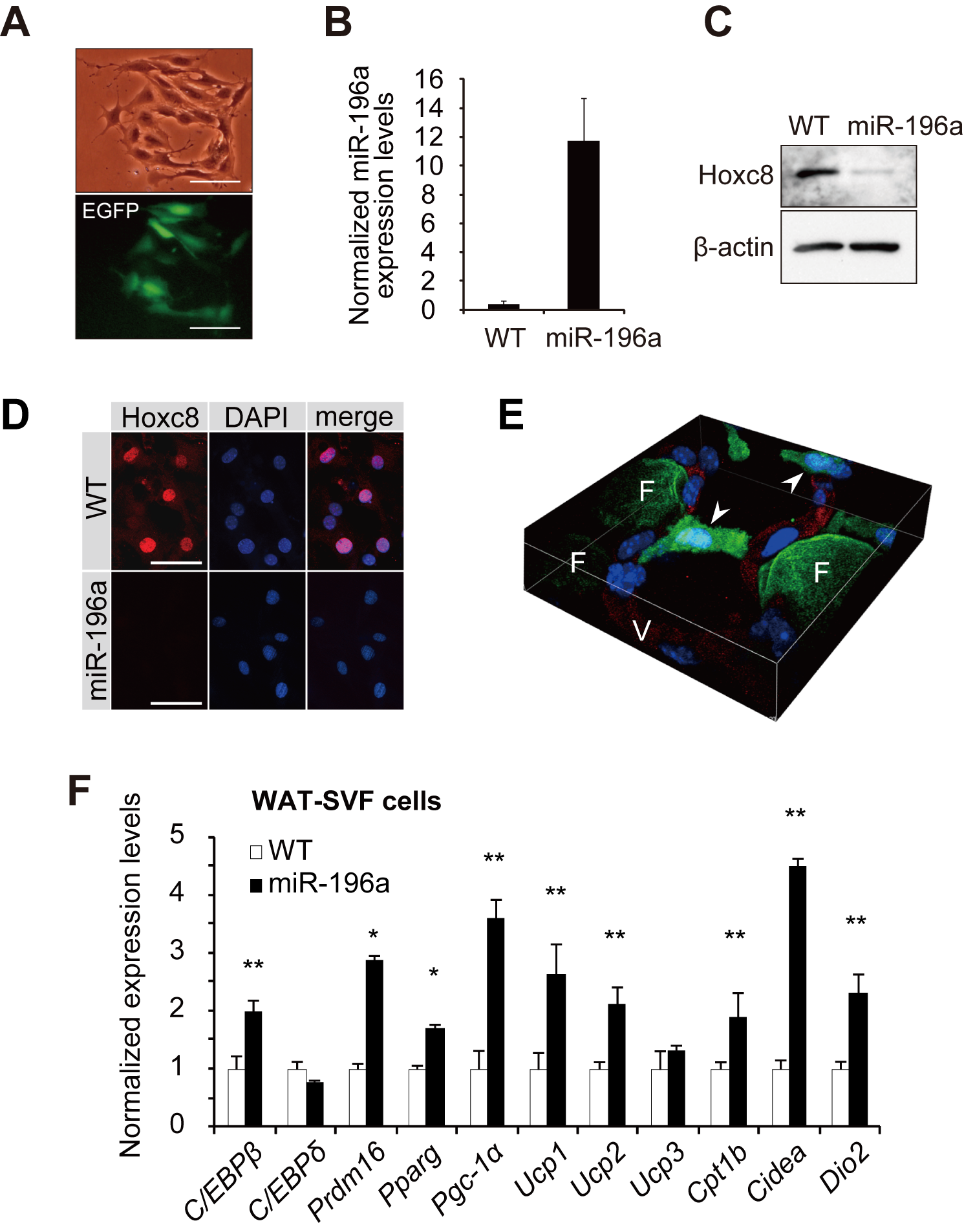

Supplement: Figure S8 — Gene expression analysis in the WAT-progenitor cells derived from the miR-196a mice. (A) The fluorescent microscopic view of the SVF cells derived from the aP2-miR-196a mice maintained without adipogenic induction. The scale bar indicates 100 µm. (B) The miR-196a expression levels in the WAT-progenitor cells derived from inguinal WAT of the WT and miR-196a mice. Data were normalized to U6. (C,D) The Western blot (C) and immunofluorescence (D) analysis of Hoxc8 in the WAT-progenitor cells. The scale bars indicate 30 µm. (E) A confocal 3-D image of an inguinal WAT from a miR-196a mouse. The vasculature and nuclei were visualized using anti-CD31 antibody and DAPI, respectively. V, vasculature; F, fat cells. (F) The gene expression analysis in WAT-progenitor cells induced to undergo adipogenesis for 14 d. Data are presented as the mean ± SEM. * p<0.05, ** p<0.01 versus WT. (TIF) [file pbio.1001314.s008.tif]

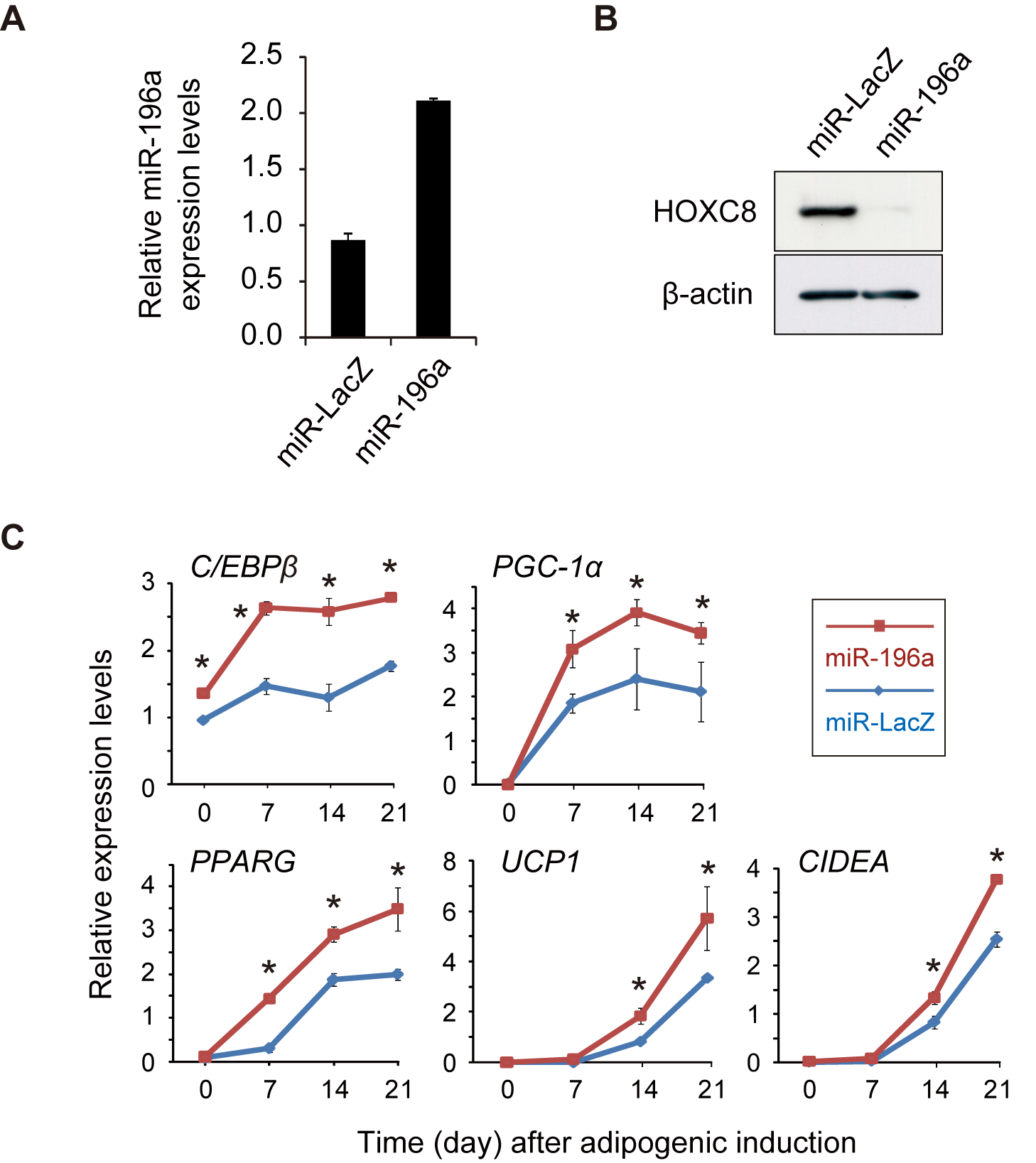

Supplement: Figure S9 — miR-196a functions in a cell-autonomous manner. (A) The qRT-PCR analysis of miR-196a in human WAT-SVF cells transduced with lentiviral vector-encoded miR-196a. The results are normalized to U6. (B) Immunoblots of HOXC8 in human WAT-SVF cells transduced with miR-196a or control miR-LacZ. β-actin served as a loading control. (C) The qRT-PCR analysis of brown fat genes in human WAT-SVF cells transduced with miR-196a or control miR-LacZ followed by adipogenic induction. All data are presented as means ± SEM. * p<0.05. (TIF) [file pbio.1001314.s009.tif]

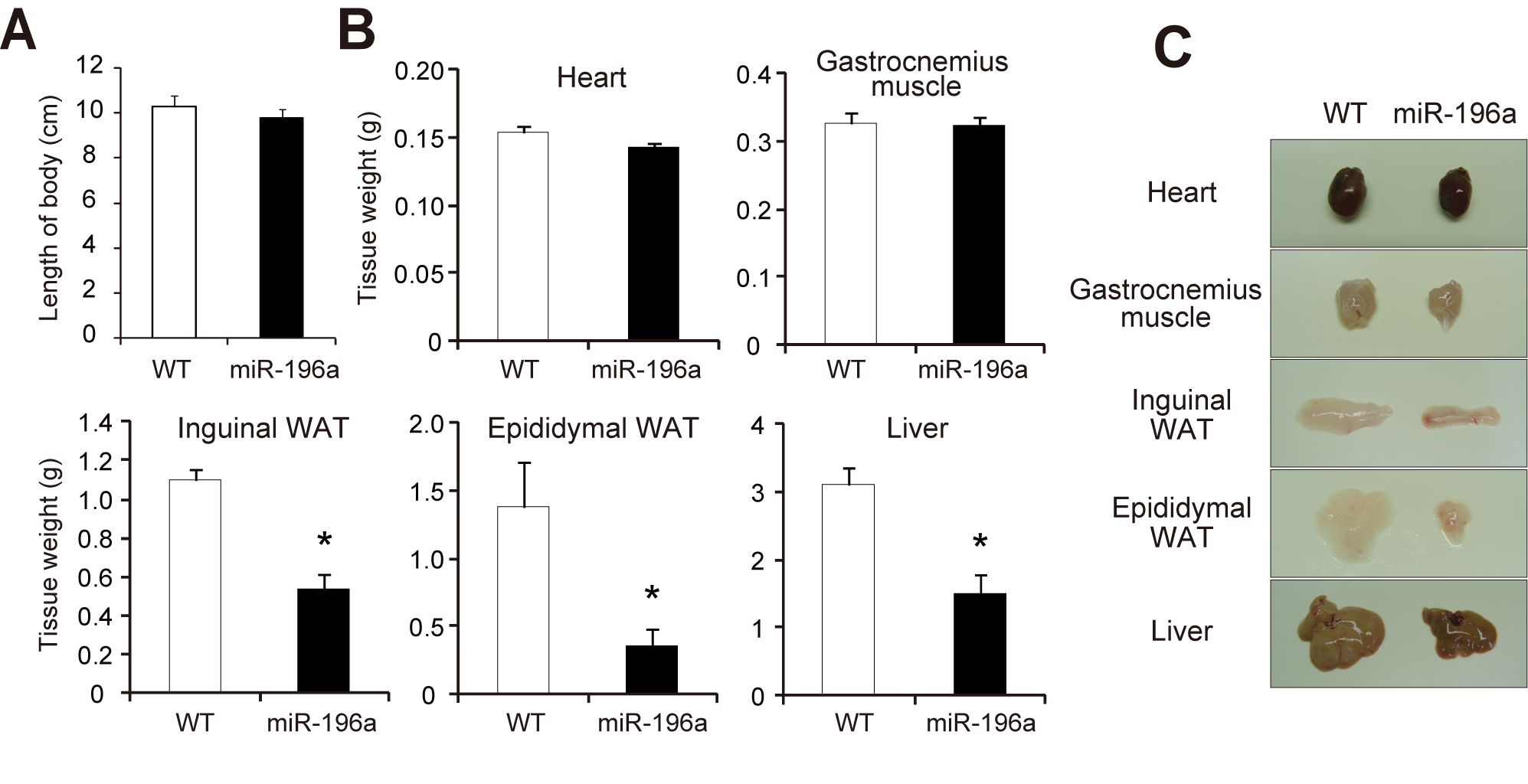

Supplement: Figure S11 — The weight reduction in the miR-196a mice is attributable to a reduced fat accumulation. (A) Body length does not differ significantly between the WT and miR-196a mice (n = 6). (B) The organ weights for the WT and miR-196a mice fed a high-fat diet for 16 wk (n = 3). The weight of the inguinal fat, epididymal WAT and liver is significantly lower in the miR-196a mice than in the WT mice. The WT mice exhibit more severe fatty livers than the miR-196a mice. All data are presented as means ± SEM. * p<0.05. (C) The appearance of the organs from the WT and miR-196a mice fed a high-fat diet for 16 wk. (TIF) [file pbio.1001314.s011.tif]
