# Supplementary material for: Essential Role for miR-196a in Brown Adipogenesis of White Fat Progenitor Cells
Source: PLoS Biol. 2012 Apr 24;10(4):e1001314. doi: 10.1371/journal.pbio.1001314 (PMC3335871; doi:10.1371/journal.pbio.1001314)
Supplement: Text S1 — TaqMan probes and ChIP primers. (PDF) [file pbio.1001314.s014.pdf]

The TaqMan probes used in the gene-expression analyses.

Gene	HUMAN	MOUSE
Hoxc8	Hs00224073	Mn00439369
Hoxc6	-	Mn01307713
Cebpb	Hs00942496	Mn00843434
Cebpd	Hs00270931	Mn00786711
Prdm16	Hs00922674	Mn00712556
Pgc-1α	Hs01016724	Mn00447183
Pparγ	Hs01115513	Mn00440945
Ucp1	Hs00222453	Mn01244861
Ucp2	Hs01075225	Mn00627599
Ucp3	Hs01106052	Mn01163394
Cpt1b	-	Mn00487200
Dio2	Hs00255341	Mn00515664
Cidea	Hs00154455	Mn00432554
Leptin	Hs00174877	Mn00434759
ADIPOQ	Hs00605917	Mm00456425
Adipsin	Hs00157263	-
CD24	Hs00273561	-
HMGA2	Hs00171569	-
18S	Hs99999901	-
β-actin	-	Mn00607939
miR-196a	241070	
U6	001093	

The sequences of the ChIP primers.

Mouse

Gene	No.	Forward (5'-3')	Reverse (5'-3')
Prdm16	1	ACGAAGAGGATGATGAACACATT	TCATCTCCCTAGCATTGTCAGTT
	2	TGTTTCTACTGGGCGTTTCTAAG	GTTGAGAACCAACAAACTGGAAG
	3	ATTCGGCATGACTTAGAACGTAA	CTCAGGATTTTCACAAAATGGAC
	4	CCAGAGCTCCTTCTGTGTTTAGA	GAAAAGAGGAGAAAACACCCACT
	5	GAGGTTTTGCTGAGGATTACAGA	TGGAAGTTCCTTGGACATATCAT
	6	GTTGAGTCTCCAACTGGGTACAA	TCTTTCCTTGAGAAAATGTCAGG
	7	CCACTGATATTTTATCCGCAGAG	GTTCACTCCCATGTCTACCTTTG
	8	CGTTGCCCACTTAAAACATAGTC	ACAAACTGACTGGGGAAGTAACA
Cebpb	1	CTCACTCTCTGCCTTCCTGGAG	CCAACACTGGTGACTATTCTTCC
	2	ACCCCAGCTCAGCAGATAAC	CTAGGTGGGCCACTCAAGC
	3	GGTCTTAGGAAGGCATCGTACTT	GGTGAGTCACTTTGCTTCTGAGT
	4	CGTTTCGAGCATTAAAGTGAAGA	CAAAATCTCGCATAAATCCAAAG
Pparγ	1	CTGGCGAGACAATGTAGCAA	TGGCACTGTCCTGACTGAGA
	2	ATGGGTGAAACTCTGGGAGA	TTGCAGCAACATCAGGAATG
	3	TTTTGTTTTTAAATGCTTTTTCACAG	CGCTGACAACGTGTTCCTT
Ucp1	1	CCAAGTGAAGACTGGGTACAAAC	CTGCTTTTTCTGGACTCATCTGT
	2	TCCATCTAAAGCTTGTCTTTTCG	GATTTCAAAGAACTTCCCTTGCT
	3	TAGTCCTAGCACTGGGAAGACAG	CCTTCCAACTTGCTTTGTTCTTA
	4	GCTCACAGACTGCCGTTTATTAT	CAGCCTGGTCTACAGAGTGAGTT
Cpt1b		AGGTTTGCTTCTGTCGGTGTAT	CTATCTGTTTTCTCCACCCCAAT
H1foo		AGGCTAGCAGTAGTCTGGATCAG	ACTGTGTCCTACCTACCTGACGAG

Human

Gene	No.	Forward (5'-3')	Reverse (5'-3')
OPN	1	TTCCCCCTACCAAATGTTCA	AAAGTGTCAGCTTCCTTTTGC
CEBPB	1	TTTCGAAGTTGATGCAATCG	CAACAAGCCCGTAGGAACAT
	2	CTTTTGGGGGCAGTAATTGG	CACACACAACCCCCTACACA
	3	CACACAGGGTCTGCTTGAGA	GAGGCTCCGGAATCTCTTCT
	4	TGGCACGCAAGAAGTGTTAT	GGTCCAGTGTTTACCAGATGC
H1FOO	1	ACAACACAGGTGACCCCAAT	CTGTGTAAGCTTGGGGGTGT
